# Supplementary material for: Current practices in studies applying the target trial emulation framework: a protocol for a systematic review
Source: BMJ Open. 2023 Jun 27;13(6):e070963. doi: 10.1136/bmjopen-2022-070963 (PMC10410979; doi:10.1136/bmjopen-2022-070963)
Supplement: Supplementary data [file bmjopen-2022-070963supp001.pdf]

Appendix

Key terms, acronyms, and abbreviations

Key term	Description
Hypothetical target trial	The open-label randomised controlled trial that, if conducted, serves as the "gold standard" with regards to evidence for treatment effectiveness.
Target trial emulation	A framework describing the application of randomised controlled trial design principles to emulate the results of a hypothetical target trial using observational data as the primary data source.
Target trial emulation study	A study applying the target trial emulation framework to estimate causal treatment effect(s) and evaluate the risk-benefit of one or more therapies.
Hypothetical target trial specification	An explicit specification of the design of the hypothetical target trial with respect to eligibility criteria, treatment strategy, assignment procedure, follow-up, primary outcome definition, the causal contrast of interest, and a statistical analysis plan.
Target trial emulation specification	An explicit specification of the design of the target trial emulation study that attempts to mimic the hypothetical target trial specification.
Study protocol	A document that details the objectives, design, planned analyses, and reporting procedures of the target trial emulation study as a whole. The study protocol is usually completed before the target trial emulation study is carried out and may be pre-registered.
Potential outcome	An example using a binary observed treatment A and binary observed outcome Y is provided.<br><br>A potential outcome is a *possible* outcome a patient could experience if they were subject to some treatment and is denoted by Y^a, where the lowercase *a* represents the treatment scenario being considered. Thus, $Y^{a=1}$ is the potential outcome of the patient under the scenario had they been given the treatment and $Y^{a=0}$ is the potential outcome of the patient under the scenario had they been given the comparator. A potential outcome then becomes a *realised* outcome once the patient actually receives their treatment. Thus a patient who actually received treatment, their potential becomes realised and their observed outcome is $Y=Y^{a=1}$.
Counterfactual	The version of the potential outcome that is not realised and thus not observed. In essence, we can never observed the patient treated and untreated at the same time.

	Using the example above, because the patient *did* take treatment, the potential outcome $Y^{a=1}$ is now factual and we can observe it, but the potential outcome $Y^{a=0}$ is now counterfactual and we can never observe it.
Exchangeability	One's observed treatment is independent of one's potential outcome. In other words, the risk of the outcome Y=1 in those who received treatment group is the same as those who received the comparator had the patients in the treatment group receive the comparator instead. In practice, this means the relationship between the treatment and the outcome is un-confounded.
Consistency	If one is given treatment option A=a, then one's observed outcome should be the potential outcome under scenario A=a i.e. $Y=Y^a$. In practice, this means the treatment options being investigated are well-defined. Depending on the context, variation within a treatment arm (e.g., different routes of administration) may be acceptable provided these variations are not expected to lead to a different potential outcome.
Positivity	Every patient has a non-zero probability of being given each treatment option under investigation, given their covariates.<br><br>There are two main types of positivity violations Structural positivity may be violated when there is a definite zero probability in some subgroup of patients. For example an investigator includes patients who are contraindicated for one the treatments under investigation. Random positivity violations are situations where whilst it is possible to see variation in treatment within a subgroup, there is no variation in that subgroup in the observed data (e.g., due to sampling variability).
Non-interference	Non-interference requires that a patient's outcome is unaffected (i.e., not interfered) by the treatment given to another patient.
**Acronyms and abbreviations**	**Full title**
CI	Confidence interval
CONSORT	Consolidated Standards of Reporting Trials
EMA	European Medicines Agency
FDA	Food and Drug Administration
HR	Hazard ratio
ICE	Inter-current event
IPTW	Inverse probability of treatment weighting
ITT	Intention-to-treat
OS	Overall survival
PP	Per-protocol

PRISMA	Preferred Reporting Items for Systematic reviews and Meta-Analyses
RCT	Randomised controlled trial
RCT DUPLICATE	Randomized, Controlled Trials Duplicated Using Prospective Longitudinal Insurance Claims: Applying Techniques of Epidemiology
RECORD	REporting of studies Conducted using Observational Routinely-collected health Data
ROBINS-I	Risk Of Bias In Non-randomized Studies – of Interventions
RWD	Real world data
RWE	Real world evidence
SMRW	Standardised morbidity ratio weights
STRATOS	STRengthening Analytical Thinking for Observational Studies
STROBE	Strengthening the Reporting of Observational Studies in Epidemiology
OPTIMAL	OPerational, TechnIcal, and MethodologicAL framework
