# Supplementary material for: Current practices in studies applying the target trial emulation framework: a protocol for a systematic review
Source: BMJ Open. 2023 Jun 27;13(6):e070963. doi: 10.1136/bmjopen-2022-070963 (PMC10410979; doi:10.1136/bmjopen-2022-070963)
Supplement: Supplementary data [file bmjopen-2022-070963supp002.pdf]

Supplementary Table 1: Search terms

The adjacency search was limited to within six words of the word “trial*”. This was to capture some possible paraphrases of “target trial emulation,” ranging from "trial emulation" to “emulation of a hypothetical randomized controlled trial”. The addition of the (Hernan and Robins) search term is to allow for studies that may have referenced the 2016 paper on the trial emulation framework in the abstract. The ISRCTN registry and ClinicalTrials.gov do not allow for proximity/adjacency searching to the best of our knowledge.

**Database**	**Search terms**
Medline (via Ovid)	((duplicat* OR emulat* OR mimic*) ADJ6 trial*).mp OR (Hernan AND Robins).ab
Embase (via Ovid)	Same as above
PsycINFO (via Ovid)	Same as above
SCOPUS	TITLE-ABS-KEY((duplicat* OR emulat* OR mimic*) W/6 trial*) OR ABS(Hernan AND Robins)
Web of Science	((duplicat* OR emulat* OR mimic*) NEAR/6 trial*) (Topic) OR (Hernan AND Robins) (Abstract)
Cochrane Library	(((duplicat* OR emulat* OR mimic*) NEAR/6 trial)):ti,ab,kw OR ((Hernan AND Robins)):ab
ISRCTN registry	"target trial" OR "target trial emulation" OR "trial emulation" OR "emulation of trial" OR "emulation of target trial" OR "emulating a trial" OR "emulating a target trial" OR "emulation of a randomized target trial" OR "emulating a randomized target trial" OR "emulation of a randomized trial" OR "emulating a randomized trial” OR "emulation of a randomized controlled trial" OR "emulating a randomized controlled trial" OR "emulation of RCT" OR "emulating RCT" OR "emulation of a clinical trial" OR "emulating a clinical trial" OR "trial-emulating" OR "hypothetical trial" OR "hypothetical target trial"
ClinicalTrials.gov	"target trial" OR "target trial emulation" OR "trial emulation" OR "emulation of trial" OR "emulation of target trial" OR "emulating a trial" OR "emulating a target trial"
