# Supplementary material for: Current practices in studies applying the target trial emulation framework: a protocol for a systematic review
Source: BMJ Open. 2023 Jun 27;13(6):e070963. doi: 10.1136/bmjopen-2022-070963 (PMC10410979; doi:10.1136/bmjopen-2022-070963)
Supplement: Supplementary data [file bmjopen-2022-070963supp003.pdf]

Supplementary Table 2: Key study features to be extracted

Level	Entire study	Target trial emulation framework	
	Target trial emulation study	Hypothetical target trial specification	Target trial emulation specification
Pre-specification	• Pre-registration ID or URL<br>• Pre-registered components: research question; eligibility criteria; treatment strategy; assignment procedure; time zero definition; follow-up period; primary outcome; causal contrasts; statistical analysis plan.		
Research meta	• Research objective<br>• Specialty area<br>• Author<br>• Year of publication<br>• Country		
Data source	• Name of observational data<br>• Type of observational data<br>• Fit-for-purpose assessment		
Target population	• Target population of interest	• Eligibility criteria	• Eligibility criteria
Treatment		• Treatment/therapy<br>• Control/comparator<br>• Grace period from eligibility to treatment initiation	• Treatment/therapy<br>• Control/comparator<br>• Grace period from eligibility to treatment initiation<br>• Time-(in)varying treatment
Assignment procedure		• Hypothetical randomisation strategy	• Method to mimic randomisation<br>• Instrumental variable or confounder selection criteria<br>• Directed acyclic graph
Baseline definition		• Time zero definition	• Time zero definition<br>• Strategy for multiple possible time zeroes<br>• Duration of each "trial's" enrolment period, if strategy is a nested trial design

Follow-up period		• Length<br>• Final visit date<br>• Censoring rules	• Length<br>• Final visit date<br>• Censoring rules
Outcome		• Name of primary outcome<br>• Operational definition	• Name of primary outcome<br>• Operational definition
Causal contrast		• ITT<br>• PP<br>• Estimand framework<br>• Hypothetical inter-current events<br>• Handling inter-current events	• Observational analogue of ITT<br>• Observational analogue of PP<br>• Estimand framework<br>• Observed inter-current events<br>• Handling observed inter-current events
Analysis		• Primary analysis method<br>• Model<br>• Adjustments	• Primary analysis method<br>• Model<br>• Handling of informative censoring<br>• Handling of missing data<br>• Assessment of causal inference assumptions
Results	• Flow diagram		
Discussion	• Design limitations limiting perfect emulation of hypothetical trial<br>• Biases limiting perfect emulation of hypothetical trial<br>• Causal inference assumptions		
Dissemination	• Published in peer-reviewed journal or archived in non-peer reviewed repository<br>• Conference material		
Others	• Reporting guideline(s) used<br>• Deviations from study protocol<br>• Future research recommendations		
