# Supplementary material for: Current practices in studies applying the target trial emulation framework: a protocol for a systematic review
Source: BMJ Open. 2023 Jun 27;13(6):e070963. doi: 10.1136/bmjopen-2022-070963 (PMC10410979; doi:10.1136/bmjopen-2022-070963)
Supplement: Author's manuscript [file bmjopen-2022-070963.draft_revisions.pdf]

BMJ Open is committed to open peer review. As part of this commitment we make the peer review history of every article we publish publicly available.

When an article is published we post the peer reviewers' comments and the authors' responses online. We also post the versions of the paper that were used during peer review. These are the versions that the peer review comments apply to.

The versions of the paper that follow are the versions that were submitted during the peer review process. They are not the versions of record or the final published versions. They should not be cited or distributed as the published version of this manuscript.

BMJ Open is an open access journal and the full, final, typeset and author-corrected version of record of the manuscript is available on our site with no access controls, subscription charges or pay-per-view fees (http://bmjopen.bmj.com).

If you have any questions on BMJ Open's open peer review process please email info.bmjopen@bmj.com

BMJ Open

Current practices in studies applying the target trial emulation framework: A protocol for a systematic review

Journal:	*BMJ Open*
Manuscript ID	bmjopen-2022-070963
Article Type:	Protocol
Date Submitted by the Author:	09-Dec-2022
Complete List of Authors:	Bigirumurame, Theophile; Newcastle University<br>Hiu, Shaun; Newcastle University<br>Wason, James; Newcastle University<br>Teare, Dawn; Newcastle University<br>Bryant, Andrew; Newcastle University
Keywords:	STATISTICS & RESEARCH METHODS, Diabetes & endocrinology < INTERNAL MEDICINE, Coronary heart disease < CARDIOLOGY

SCHOLARONE™
Manuscripts

TITLE: Current practices in studies applying the target trial emulation framework: A protocol for a systematic review

Short title: Practices in trial emulation

Authors: Theophile Bigirumurame[1], Shaun Kuan Wei Hiu[1], M. Dawn Teare[1], James Wason[1], Andrew Bryant[1]

[1]Population Health Sciences Institute, Newcastle University, UK

Corresponding author:
Theophile Bigirumurame
Population Health Sciences Institutes, Newcastle University,
4th Floor
Ridley Building 1
Queen Victoria Road
Newcastle upon Tyne
NE1 7RU
UK,
Email: theophile.bigirumurame@newcastle.ac.uk
Tel: +44 (0)19120 86776

Authors email addresses: Shaun.Hiu@newcastle.ac.uk, Dawn.Teare@newcastle.ac.uk, James.Wason@newcastle.ac.uk , andy.bryant@newcastle.ac.uk

Abstract:
Introduction: Randomised controlled trials (RCTs) are the "gold standard" to evaluate the efficacy and safety of treatments. Observational studies represent an alternative to estimate real-world causal effects in the absence of available RCTs. Target trial emulation is a framework for the application of RCT design principles to emulate a hypothetical open-label RCT (the hypothetical target trial) using existing observational data as the primary data source as opposed to the prospective recruitment and measurement of randomised units. The aim of this systematic review is to investigate the practices of studies applying the target trial emulation framework to evaluate the effectiveness of interventions.

Methods and analysis We will systematically search in Pubmed (incorporating Medline), Ovid (incorporating Embase), PsycINFO, SCOPUS, Cochrane Library, Web of Knowledge, the ISRCTN registry, ClinicalTrials.gov, and medRxiv for all study reports, protocols, reviews, and methodological papers which mentioned trial emulation (without time restriction). We will extract information concerning study design, data source, analysis, results, interpretation, and dissemination.

Two reviewers will perform study selection, data extraction and quality assessment. Disagreements between reviewers will be resolved by a third reviewer. A narrative approach will be used to synthesise and report qualitative and quantitative data. Reporting of the review will be informed by Preferred Reporting Items for Systematic Review and Meta-Analysis guidance (PRISMA-P).

Ethics and dissemination Ethical approval is not required as it is a protocol for a systematic review. Findings will be disseminated through peer-reviewed publications and conference presentations.

Keywords:
Trial emulation; hypothetical target trial; Target trial emulation; Target trial emulation study, Trial emulation systematic review

Word count= 2257 (excluding abstract, table text, captions, acknowledgment, funding source statement, declaration of interest statement, author contribution statement, and references).

Strengths and limitations

- This systematic review protocol follows the Preferred Reporting Items for Systematic Review and Meta-Analysis Protocols guidelines.
- This systematic review will address a gap in the current practices in studies applying the target trial emulation framework by identifying current strengths and weaknesses in how target trial emulation studies are being reported and may be an evidence-base to inform a bespoke set of reporting guidelines and risk of bias tools for target trial emulation
- This review is limited to evidence from published/archived studies
- Non-English electronic databases will not be searched. This limitation may cause language bias.

For peer review only - http://bmjopen.bmj.com/site/about/guidelines.xhtml

Introduction

Randomised controlled trials (RCTs) are the "gold standard" to evaluate the efficacy and safety of treatments. Nevertheless, the external validity of RCTs may be limited due the restricted diversity of eligible patients. For instance, very old patients or patients with multiple comorbidities are often not eligible for RCTs [1, 2].

Observational studies represent an alternative to estimate real-world causal effects in the absence of available RCTs. Reasons to conduct observational studies instead of RCTs may include feasibility, ethical considerations, reduced associated costs, and greater time efficiency. Whilst the use of observational studies to attempt to estimate causal effects is not a new phenomenon – the practice of which dates back as early as Rubin's work [3] on counterfactual theory – the application of RCT design principles to overcome biases in and improve the quality of observational research has been a relatively new development.

Target trial emulation is a framework for the application of RCT design principles to emulate a hypothetical open-label RCT (the hypothetical target trial) using existing observational data as the primary data source as opposed to the prospective recruitment and measurement of randomised units [4]. Because treatment assignment is neither blind nor random, valid causal treatment effects are estimated if the identification principles of causal inference are satisfied [4-8]. These include exchangeability, consistency, positivity, and non-interference [7].

A study employing target trial emulation is characterised by an explicit description of the hypothetical target trial on several design components: the eligibility criteria, treatment strategy, assignment procedure, follow-up, primary outcome definition, the causal contrast of interest, and a statistical analysis plan [4]. Additionally, an accompanying target trial emulation specification is described and makes explicit how the hypothetical target trial is being emulated along the aforementioned design elements. Stating both the hypothetical target trial and target trial emulation specifications has important benefits. It assists investigators in avoiding biases and pitfalls that are common to the analysis of observational data [9, 10], and permits the sceptical reader to judge if the design of the hypothetical target trial was sensible and if emulation was satisfactory; for example, the eligibility criteria may not be faithfully emulated owing to limitations with the observational data.

Target trial emulation studies can be credible sources of real-world evidence (RWE) to support regulatory decisions in the absence of RCTs, for instance within the Food and Drug Administration's Real-World Evidence Program [11] and the European Medicines Agency's OPerational, TechnIcal, and MethodologicAL framework (OPTIMAL) [12]. Furthermore, with the increasing availability of large-scale real-world data (RWD) especially those seen in research cohorts, registry, hospital, and general practitioner databases, there is impetus for increased popularity of the application of target trial emulation.

To our knowledge, no study has reviewed the extant work applying the target trial emulation framework. We argue that a review of this nature is needed for several reasons that have implications for the continued growth of target trial emulation.

For peer review only - http://bmjopen.bmj.com/site/about/guidelines.xhtml

First, a systematic review (SR) that reports on the design, conduct, and reporting of target trial emulation studies will help facilitate new research hoping to utilise the methodology. Investigators seeking to conduct their own target trial emulation study may draw guidance from what is currently published, and so a systematic review of the literature serves as a convenient repository of knowledge to new investigators who may be unfamiliar with terminology, choices of designs and analyses, and reporting practices. An additional benefit of the review to both new and experienced investigators is the increased awareness of available datasets that may be pertinent to their field of expertise.

Second, target trial emulation studies attempt to mimic RCTs and thus the Consolidated Standards of Reporting Trials (CONSORT) checklist [13] may be a valid guideline for reporting standards. However, given that target trial emulation studies are necessarily observational studies, the Strengthening the Reporting of Observational Studies in Epidemiology (STROBE) guidelines also apply [14]. Thus, it is essential to capture what (or whether) established reporting guideline(s) have been adopted within the existing trial emulation literature, so that future studies may follow suit. Furthermore, with the recent movement towards the language of the estimand framework in clinical trials [15], it may be useful to capture whether the target trial emulation literature has also displayed such a shift. It may also be that existing checklists are not sufficient to capture all the nuances that exist in target trial emulation. For example, explicit statements on quality assessment such as how the data have been evaluated and judged to be fit-for-purpose for trial emulation are not expected by existing guidelines. Thus, any available reports on data quality cleaning and checks may be selective. Clear reporting of these steps is critical especially for regulatory purposes that may require relevance assessments demonstrating fit-for-purpose of the RWD [11]. Additionally, pre-registration is not expected for observational studies but is expected for RCTs. At present there is uncertainty over whether pre-registration should be mandatory, or at least encouraged, for target trial emulation studies.

Third, it may be useful to understand how published studies have contextualised the interpretation of their estimated treatment effects in relation to the idealised hypothetical target trial. Recent findings from the Randomized, Controlled Trials Duplicated Using Prospective Longitudinal Insurance Claims: Applying Techniques of Epidemiology (RCT DUPLICATE) initiative compared results of RCTs with their emulation counterparts and found that they do not necessarily agree because perfect emulation may not be possible [16]. This may be due to, but not limited to, differences in treatment adherence rates between RCTs and real-world practice, partial emulation of eligibility criteria due to database limitations, measurement error in the primary outcome, and the challenges associated with emulating a placebo add-on arm. Although not every study applying the target trial emulation framework will be able to make comparisons with results from an actual RCT, it is still crucial for authors to postulate on the factors that may strengthen or qualify their interpretation of their results.

Lastly, our SR will help identify current strengths and weaknesses in how target trial emulation studies are being reported and may be an evidence-base to inform a bespoke set of reporting guidelines and risk of bias tools for target trial emulation.

For peer review only - http://bmjopen.bmj.com/site/about/guidelines.xhtml

Research objectives

The objective of this SR is to investigate the practices of studies applying the target trial emulation framework to evaluate the risk-benefit of interventions. We aim to examine multiple facets of the study process, specifically pre-specification, study design, data source, analysis, results, interpretation, and dissemination (Table 1).

For peer review only - http://bmjopen.bmj.com/site/about/guidelines.xhtml

Table 1: Key research questions

Component	Key questions
Pre-registration	a. Was a study protocol developed and/or published/archived in a repository?<br>b. Which components of the target trial emulation study were pre-registered?
Study design	a. Was the research objective clearly stated?<br>b. Were stakeholders included in the study design process?<br>c. Was a causal directed acyclic graph reported?
Population	a. What was the population of interest?
Eligibility criteria	a. Were there differences in the eligibility criteria between the target trial emulation specification and hypothetical target trial specification?
Treatment strategy	a. What was the intervention and what was the comparator?<br>b. How were treatment groups defined?<br>c. Was a grace period applied from the point of eligibility to treatment initiation? Was the length justified?<br>d. Was there a washout period prior to treatment initiation (i.e. a period between the cessation of one or more therapies and the initiation of the treatment under investigation)?
Assignment procedure	a. How was the randomisation strategy in the hypothetical target trial mimicked?<br>b. How were confounders selected?
Time zero and follow up	a. How did they define the baseline visit (time zero)?<br>b. How did they define the follow-up period?<br>c. If there are multiple possible time-zeros (i.e. patient met trial eligibility criteria at several points in the observational data), how was this handled?<br>d. What were the censoring rules, if the outcome was time-to-event?

For peer review only - http://bmjopen.bmj.com/site/about/guidelines.xhtml

Primary outcome	a. What was the primary outcome?<br>b. How was the primary outcome operationally defined?<br>c. Was the primary outcome clearly defined before any data were analysed?<br>d. Was a minimally clinically important difference reported?
Causal contrast	a. What were causal contrasts: intention to treat (ITT) vs Per-protocol (PP)?<br>b. How did ITT differ from PP?<br>c. Was estimand framework used?<br>d. What were the estimand(s), if reported?<br>e. What were the inter-current event(s), if reported?
Analysis methods	a. Which statistical methods were used?<br>b. Were assumptions of causal inference mentioned and justified?<br>c. Were sensitivity analyses performed?<br>d. How did they handle different types of confounders and biases?<br>e. If propensity score methods were used, how were these reported?<br>f. If instrumental variable analysis was used, how was the variable argued to be a valid instrumental variable?
Sample size	a. How was the sample size derived?<br>b. Were simulations performed?
Data source	a. What data source was used?<br>b. Was there information on cost of the data or being publicly available?<br>c. Were multiple data sources used to reproduce their results?<br>c. Was any assessment done to check that the data were fit for purpose?<br>d. Were any limitations of the dataset reported that limit extent of emulation?
Results	a. Was a flow diagram reported detailing how the final sample size was derived from the observational data?<br>b. Was the propensity score model reported?

For peer review only - http://bmjopen.bmj.com/site/about/guidelines.xhtml

	c. Were the results of the primary and sensitivity analyses reported?
Interpretation	a. How were estimated treatment effects interpreted, specifically within the context of the hypothetical target trial?
Dissemination	a. Were results published and/or archived?
Others	a. Were any reporting checklists used?<br>b. Was the study purpose clearly stated?<br>c. Were there deviations from the original study protocol?<br>d. Were deviations from the original study protocol reported?

Methods

Database search

The following electronic databases will be searched: Pubmed (incorporating Medline), Ovid (incorporating Embase), PsycINFO, SCOPUS, Cochrane Library, Web of Knowledge, the ISRCTN registry, ClinicalTrials.gov, and medRxiv. The following search terms will be used:

("target" OR "trial") AND (emulat* OR duplicat*)

Articles will also be identified from the reference lists of the included studies, as well as a Google Scholar search of studies that have cited the included studies. The search will be limited to articles published in English only, and no publication date limits will be applied.

Study inclusion and exclusion criteria

Our definition of a target trial emulation study follows from Hernán & Robins [4]:

1. Presence of a hypothetical target trial specification which explicitly describes the trial design components of eligibility criteria, treatment strategy, assignment procedure, follow-up, primary outcome definition, the causal contrast of interest, and a statistical analysis plan;
2. Presence of a target trial emulation specification which explicitly describes how the hypothetical target trial is being emulated. Example can be seen in [9];
3. Use of at least one observational data source.

Inclusion criteria are as follows: i) meets study definition of target trial emulation study; ii) peer-reviewed reports, protocols (published or archived), study registrations, and conference presentations on original research studies that used/plan to use the trial emulation, from all fields of research; and iii) available in English language. Studies which sought to replicate RCT findings with RWD will also be included since the RCT protocol contains all the information needed in a hypothetical target trial specification and the target trial emulation specification may be inferred from the methods section.

Exclusion criteria are: a) conference abstracts, book chapters, opinions or commentaries, purely qualitative papers, systematic reviews, and meta-analyses; b) conceptual/methodological papers that do not use data; and c) non-human subjects. The reference sections of these papers will be checked to identify eligible papers.

Selection of studies for inclusion in the review

TB and SKWH will read all titles/and or abstracts retrieved from the database search process and eliminate obviously irrelevant records. The full text of the remaining possibly relevant studies will then be obtained and read, with each record classified as clearly relevant (meets all inclusion criteria), clearly irrelevant, or insufficient information to decide. For those cases with insufficient information, corresponding author(s) will be contacted for further information to make a final decision on relevance. In the instance that the full text of a possibly relevant study cannot be retrieved, the corresponding author(s) will be contacted and if no reply is received,

For peer review only - http://bmjopen.bmj.com/site/about/guidelines.xhtml

the record will be excluded from the final review. Disagreements on eligibility of studies will be arbitrated by a third co-author. References will be managed on Rayyan.

Data extraction and management

TB and SKWH will extract data from all included records according to a bespoke data capture form (DCF) developed in Excel and designed around the key research questions. The DCF will be designed to help review co-authors elicit the required information from the study text with specific questions and prompts (Table 2). Pre-defined responses will be available for all straightforward yes/no-type questions and certain multiple-choice questions. The multiple-choice questions with pre-defined responses will be those in which the investigators have prior knowledge on a list of possible responses; lists that are judged to be non-exhaustive will be supplemented by an 'Other' response. For questions without this prior knowledge, we will ask the co-author extracting the information to copy the appropriate text verbatim from the paper. The text data will then be subjected to a coding process (see Data coding section).

Version 1.0 of the DCF will be developed by adapting information from existing reporting frameworks that are judged to be applicable to target trial emulation. These are the:

1. CONSORT [13];
2. STROBE (combined checklist) [14];
3. REporting of studies Conducted using Observational Routinely-collected health Data (RECORD) [17];
4. The estimand framework [15, 18];
5. Risk Of Bias In Non-randomized Studies – of Interventions (ROBINS-I) assessment tool [19]; and
6. The STRengthening Analytical Thinking for Observational Studies (STRATOS) publications on initial data analysis [20], missing data [21], and causal inference [22].

Version 2.0 will then be piloted on 10 of the included papers and adjustments made where appropriate via discussion with additional authors (JW and MDT). Version 3.0 will then be used for all remaining papers.

Data coding

The extracted data will come in the form of close-ended choices (e.g., yes/no and other pre-defined responses), numerical data (e.g., sample size), and open-ended text.

For ease of reporting of the text data, they will be categorised into codes with respect to features of the study. A list of codes will be generated from a bottom-up process (i.e., driven by the data) via discussions between all study authors and after each author has reviewed the text data at least once. Codes will then be categorised into higher-level groups called themes. As such, codes can be regarded as levels within a theme factor.

Summarising and reporting of results

For peer review only - http://bmjopen.bmj.com/site/about/guidelines.xhtml

The current protocol adheres to the Preferred Reporting Items for Systematic reviews and Meta-Analyses–Protocol (PRISMA-P) reporting guidelines [23]. Our SR will adhere to the PRISMA reporting guidelines [24, 25].

The study characteristics will be described with respect to sample demographics, sample size, specialty area, year of publication, and data source type.

For each research question, we will describe the frequency (%) of studies displaying the relevant characteristic(s). For example, to answer the question on sample size justification, we may the frequency and percentage of studies with the following characteristics: "*None stated*", "*Based on available data*", "*Closed form equation*", and "*Statistical simulation*". The *Type of Simulation* may also be described to further elaborate on what parameters were varied in the statistical simulation – and the codes used may be derived from an analysis of text data for example.

Risk of bias assessment

No risk of bias assessment will be carried out. It is not within the scope of our objectives to render a judgment on whether the hypothetical target trial was successfully emulated or judge if the study results as credible evidence to inform the effectiveness of a treatment. The evidence being synthesised will primarily be the characteristics of the studies and reporting patterns, as opposed to their results, which themselves are reflective of the methodological quality needed for risks for bias assessments.

Conclusion

This systematic review of target trial emulation studies will give future researchers a detailed overview of how target trial emulation studies have been conducted and reported. Understanding the current strengths and weaknesses of how current studies are being reported will help inform bespoke reporting guidelines and risk of bias tools.

For peer review only - http://bmjopen.bmj.com/site/about/guidelines.xhtml

Table 2: Outline of features to be extracted

Level	Entire study	Target trial emulation framework	
	Target trial emulation study	Hypothetical target trial specification	Target trial emulation specification
Pre-specification	• Pre-registration ID or URL<br>• Pre-registered components: research question; eligibility criteria; treatment strategy; assignment procedure; time zero definition; follow-up period; primary outcome; causal contrasts; statistical analysis plan.		
Research meta	• Research objective<br>• Research question<br>• Specialty area		
Sample characteristics	• Author<br>• Year of publication<br>• Sample size<br>• Demographics: age, sex		
Data source	• Name of observational data<br>• Type of source: research cohort; registry; hospital; GP; claims<br>• Publicly available or requires cost<br>• Fit-for-purpose assessment		
Target population	• Target population of interest	• Eligibility criteria	• Eligibility criteria
Treatment		• Treatment/therapy<br>• Control/comparator<br>• Grace period from eligibility to treatment initiation	• Treatment/therapy<br>• Control/comparator<br>• Grace period from eligibility to treatment initiation
Assignment procedure		• Hypothetical randomisation strategy	• Method to mimic randomisation<br>• Instrumental variable or confounder selection criteria<br>• Directed acyclic graph
Baseline definition		• Time zero	• Time zero

			• Strategy for multiple possible time zeroes<br>• Duration of each "trial's" enrolment period, if strategy is a nested trial design
Follow-up period		• Length<br>• Final visit date<br>• Censoring rules	• Length<br>• Final visit date<br>• Censoring rules
Outcome		• Name of primary outcome<br>• Operational definition	• Name of primary outcome<br>• Operational definition
Causal contrast		• ITT<br>• PP<br>• Estimand framework<br>• Hypothetical inter-current events<br>• Handling inter-current events	• ITT<br>• PP<br>• Estimand framework<br>• Observed inter-current events<br>• Handling observed inter-current events
Analysis		• Primary analysis method<br>• Model<br>• Adjustments<br>• Sample size and clinically important difference	• Primary analysis method<br>• Model<br>• Matching method<br>• Weights<br>• Propensity score model specification<br>• Causal inference assumptions<br>• Sample size/power justification
Results	• Flow diagram<br>• Propensity score model		
Interpretation	• Design limitations limiting perfect emulation of hypothetical trial<br>• Biases limiting perfect emulation of hypothetical trial		
Dissemination	• Published and/or archived results<br>• Conference material		
Others	• Reporting guideline(s) used<br>• Deviations from study protocol<br>• Future research recommendations		

ID, identifier; ITT, intention-to-treat; PP, per-protocol; URL, uniform resource locator.

For peer review only - http://bmjopen.bmj.com/site/about/guidelines.xhtml

References

1. Kennedy-Martin T, Curtis S, Faries D, Robinson S, Johnston J. A literature review on the representativeness of randomized controlled trial samples and implications for the external validity of trial results. Trials. 2015;16:495.
2. Maringe C, Benitez Majano S, Exarchakou A, Smith M, Rachet B, Belot A, et al. Reflection on modern methods: trial emulation in the presence of immortal-time bias. Assessing the benefit of major surgery for elderly lung cancer patients using observational data. Int J Epidemiol. 2020;49:1719-29.
3. Rubin DE. Estimating causal effects of treatments in randomized and nonrandomized studies. Journal of Educational Psychology. 1974;66:688-701.
4. Hernán MA, Robins JM. Using Big Data to Emulate a Target Trial When a Randomized Trial Is Not Available. American Journal of Epidemiology. 2016;183:758-64.
5. García-Albéniz X, Hsu J, Hernán MA. The value of explicitly emulating a target trial when using real world evidence: an application to colorectal cancer screening. European Journal of Epidemiology. 2017;32:495-500.
6. Hernán MA, Alonso A, Logan R, Grodstein F, Michels KB, Willett WC, et al. Observational studies analyzed like randomized experiments: an application to postmenopausal hormone therapy and coronary heart disease. Epidemiology. 2008;19:766-79.
7. Hernán MA, Robins JM. Causal Inference: What If. Boca Raton: Chapman & Hall/CRC; 2020.
8. Lodi S, Phillips A, Lundgren J, Logan R, Sharma S, Cole SR, et al. Effect Estimates in Randomized Trials and Observational Studies: Comparing Apples With Apples. Am J Epidemiol. 2019;188:1569-77.
9. Dickerman BA, Garcia-Albeniz X, Logan RW, Denaxas S, Hernán MA. Avoidable flaws in observational analyses: an application to statins and cancer. Nat Med. 2019;25:1601-6.
10. Hernán MA, Sauer BC, Hernandez-Diaz S, Platt R, Shrier I. Specifying a target trial prevents immortal time bias and other self-inflicted injuries in observational analyses. J Clin Epidemiol. 2016;79:70-5.
11. US Food and Drug Administration. Framework for FDA's Real-World Evidence Program. 2018.
12. Cave A, Kurz X, Arlett P. Real-World Data for Regulatory Decision Making: Challenges and Possible Solutions for Europe. Clin Pharmacol Ther. 2019;106:36-9.
13. Schulz KF, Altman DG, Moher D. CONSORT 2010 Statement: Updated Guidelines for Reporting Parallel Group Randomized Trials. Annals of Internal Medicine. 2010;152:726-32.
14. von Elm E, Altman DG, Egger M, Pocock SJ, Gøtzsche PC, Vandenbroucke JP. The Strengthening the Reporting of Observational Studies in Epidemiology (STROBE) Statement: Guidelines for Reporting Observational Studies. Annals of Internal Medicine. 2007;147:573-7.
15. Ratitch B, Bell J, Mallinckrodt C, Bartlett JW, Goel N, Molenberghs G, et al. Choosing Estimands in Clinical Trials: Putting the ICH E9(R1) Into Practice. Ther Innov Regul Sci. 2020;54:324-41.
16. Franklin JM, Patorno E, Desai RJ, Glynn RJ, Martin D, Quinto K, et al. Emulating Randomized Clinical Trials With Nonrandomized Real-World Evidence Studies: First Results From the RCT DUPLICATE Initiative. Circulation. 2021;143:1002-13.
17. Benchimol EI, Smeeth L, Guttmann A, Harron K, Moher D, Petersen I, et al. The REporting of studies Conducted using Observational Routinely-collected health Data (RECORD) Statement. PLOS Medicine. 2015;12:e1001885.
18. Lipkovich I, Ratitch B, Mallinckrodt CH. Causal Inference and Estimands in Clinical Trials. Statistics in Biopharmaceutical Research. 2020;12:54-67.
19. Sterne JAC, Hernán MA, Reeves BC, Savović J, Berkman ND, Viswanathan M, et al. ROBINS-I: a tool for assessing risk of bias in non-randomised studies of interventions. BMJ. 2016;355:i4919.
20. Huebner M, le Cessie S, Schmidt CO, Vach W. A contemporary conceptual framework for initial data analysis. Observational Studies. 2018;4:171-92.

For peer review only - http://bmjopen.bmj.com/site/about/guidelines.xhtml

21. Lee KJ, Tilling KM, Cornish RP, Little RJA, Bell ML, Goetghebeur E, et al. Framework for the treatment and reporting of missing data in observational studies: The Treatment And Reporting of Missing data in Observational Studies framework. J Clin Epidemiol. 2021;134:79-88.
22. Goetghebeur E, le Cessie S, De Stavola B, Moodie EE, Waernbaum I, on behalf of" the topic group Causal Inference of the Si. Formulating causal questions and principled statistical answers. Stat Med. 2020;39:4922-48.
23. Moher D, Shamseer L, Clarke M, Ghersi D, Liberati A, Petticrew M, et al. Preferred reporting items for systematic review and meta-analysis protocols (PRISMA-P) 2015 statement. Syst Rev. 2015;4:1.
24. Moher D, Liberati A, Tetzlaff J, Altman DG, Group P. Preferred reporting items for systematic reviews and meta-analyses: the PRISMA statement. Int J Surg. 2010;8:336-41.
25. Page MJ, McKenzie JE, Bossuyt PM, Boutron I, Hoffmann TC, Mulrow CD, et al. The PRISMA 2020 statement: An updated guideline for reporting systematic reviews. Int J Surg. 2021;88:105906.

For peer review only - http://bmjopen.bmj.com/site/about/guidelines.xhtml

ACKNOWLEDGMENT

We are grateful for the funding provided by the National Institute for Health Research (NIHR).

CONTRIBUTORSHIP STATEMENT

Design of the protocol- TB, SKWH. Draft of the manuscript -TH, SKWH. Review and final approval of the manuscript –TH, SKWH, JM, MD, AB.

FUNDING STAEMENT

TB is funded by the NIHR Applied Research Collaboration North East and North Cumbria (Grant/Award Number: NIHR2001). SKWH (Pre-Doctoral Fellowship, NIHR302746) is funded by the National Institute for Health Research (NIHR) for this research project. The views expressed in this publication are those of the author(s) and not necessarily those of the NIHR, National Health Service (NHS), or the UK Department of Health and Social Care.

CONFLICTS OF INTERESTS STATEMENT

None declared

DATA AVAILABILITY STATEMENT

All data relevant to the study protocol are included in the article.

ETHICAL APPROVAL

Not required

ORCID:

Theophile Bigirumurame: https://orcid.org/0000-0003-2387-4918

Shaun Kuan Wei Hiu: https://orcid.org/0000-0003-1699-4348

M. Dawn Teare: https://orcid.org/0000-0003-3994-0051

James Wason: https://orcid.org/0000-0002-4691-126X

Andrew Bryant: https://orcid.org/0000-0003-4351-8865

Appendix

Key terms, acronyms, and abbreviations

Key term	Description
Hypothetical target trial	The open-label randomised controlled trial that, if conducted, serves as the "gold standard" with regards to evidence for treatment effectiveness.
Target trial emulation	A framework describing the application of randomised controlled trial design principles to emulate the results of a hypothetical target trial using observational data as the primary data source.
Target trial emulation study	A study applying the target trial emulation framework to estimate causal treatment effect(s) and evaluate the risk-benefit of one or more therapies.
Hypothetical target trial specification	An explicit specification of the design of the hypothetical target trial with respect to eligibility criteria, treatment strategy, assignment procedure, follow-up, primary outcome definition, the causal contrast of interest, and a statistical analysis plan.
Target trial emulation specification	An explicit specification of the design of the target trial emulation study that attempts to mimic the hypothetical target trial specification.
Study protocol	A document that details the objectives, design, planned analyses, and reporting procedures of the target trial emulation study as a whole. The study protocol is usually completed before the target trial emulation study is carried out and may be pre-registered.
**Acronyms and abbreviations**	**Full title**
CI	Confidence interval
CONSORT	Consolidated Standards of Reporting Trials
EMA	European Medicines Agency
FDA	Food and Drug Administration
HR	Hazard ratio
ICE	Inter-current event
IPTW	Inverse probability of treatment weighting
ITT	Intention-to-treat
OS	Overall survival
PP	Per-protocol
PRISMA	Preferred Reporting Items for Systematic reviews and Meta-Analyses
RCT	Randomised controlled trial
RCT DUPLICATE	Randomized, Controlled Trials Duplicated Using Prospective Longitudinal Insurance Claims: Applying Techniques of Epidemiology

For peer review only - http://bmjopen.bmj.com/site/about/guidelines.xhtml

RECORD	REporting of studies Conducted using Observational Routinely-collected health Data
ROBINS-I	Risk Of Bias In Non-randomized Studies – of Interventions
RWD	Real world data
RWE	Real world evidence
SMRW	Standardised morbidity ratio weights
STRATOS	STRengthening Analytical Thinking for Observational Studies
STROBE	Strengthening the Reporting of Observational Studies in Epidemiology
OPTIMAL	OPerational, TechnIcal, and MethodologicAL framework

For peer review only - http://bmjopen.bmj.com/site/about/guidelines.xhtml

Reporting checklist for protocol of a systematic review and meta analysis.

Based on the PRISMA-P guidelines.

Instructions to authors

Complete this checklist by entering the page numbers from your manuscript where readers will find each of the items listed below.

Your article may not currently address all the items on the checklist. Please modify your text to include the missing information. If you are certain that an item does not apply, please write "n/a" and provide a short explanation.

Upload your completed checklist as an extra file when you submit to a journal.

In your methods section, say that you used the PRISMA-Preporting guidelines, and cite them as:

Moher D, Shamseer L, Clarke M, Ghersi D, Liberati A, Petticrew M, Shekelle P, Stewart LA. Preferred Reporting Items for Systematic Review and Meta-Analysis Protocols (PRISMA-P) 2015 statement. Syst Rev. 2015;4(1):1.

		Reporting Item	Page Number
**Title**			
Identification	#1a	Identify the report as a protocol of a systematic review	1
Update	#1b	If the protocol is for an update of a previous systematic review, identify as such	n/a

For peer review only

**Registration**			
	#2	If registered, provide the name of the registry (such as PROSPERO) and registration number	n/a methodological review
**Authors**			
Contact	#3a	Provide name, institutional affiliation, e-mail address of all protocol authors; provide physical mailing address of corresponding author	1
Contribution	#3b	Describe contributions of protocol authors and identify the guarantor of the review	16
**Amendments**			
	#4	If the protocol represents an amendment of a previously completed or published protocol, identify as such and list changes; otherwise, state plan for documenting important protocol amendments	n/a first version
**Support**			
Sources	#5a	Indicate sources of financial or other support for the review	16
Sponsor	#5b	Provide name for the review funder and / or sponsor	16
Role of sponsor or funder	#5c	Describe roles of funder(s), sponsor(s), and / or institution(s), if any, in developing the protocol	n/a
**Introduction**			

For peer review only

Rationale	#6	Describe the rationale for the review in the context of what is already known	3
Objectives	#7	Provide an explicit statement of the question(s) the review will address with reference to participants, interventions, comparators, and outcomes (PICO)	n/a methodological review
**Methods**			
Eligibility criteria	#8	Specify the study characteristics (such as PICO, study design, setting, time frame) and report characteristics (such as years considered, language, publication status) to be used as criteria for eligibility for the review	9
Information sources	#9	Describe all intended information sources (such as electronic databases, contact with study authors, trial registers or other grey literature sources) with planned dates of coverage	9
Search strategy	#10	Present draft of search strategy to be used for at least one electronic database, including planned limits, such that it could be repeated	9
Study records - data management	#11a	Describe the mechanism(s) that will be used to manage records and data throughout the review	10
Study records - selection process	#11b	State the process that will be used for selecting studies (such as two independent reviewers) through each phase of the review (that is, screening, eligibility and	10

For peer review only

		inclusion in meta-analysis)	
Study records - data collection process	#11c	Describe planned method of extracting data from reports (such as piloting forms, done independently, in duplicate), any processes for obtaining and confirming data from investigators	10
Data items	#12	List and define all variables for which data will be sought (such as PICO items, funding sources), any pre-planned data assumptions and simplifications	6,7,8
Outcomes and prioritization	#13	List and define all outcomes for which data will be sought, including prioritization of main and additional outcomes, with rationale	n/a methodological review
Risk of bias in individual studies	#14	Describe anticipated methods for assessing risk of bias of individual studies, including whether this will be done at the outcome or study level, or both; state how this information will be used in data synthesis	n/a methodological review
Data synthesis	#15a	Describe criteria under which study data will be quantitatively synthesised	n/a methodological review
Data synthesis	#15b	If data are appropriate for quantitative synthesis, describe planned summary measures, methods of handling data and methods of combining data from studies, including any planned exploration of consistency (such as I2, Kendall's τ)	n/a methodological review

For peer review only

Data synthesis	#15c	Describe any proposed additional analyses (such as sensitivity or subgroup analyses, meta-regression)	n/a methodological review
Data synthesis	#15d	If quantitative synthesis is not appropriate, describe the type of summary planned	Descriptive summary
Meta-bias(es)	#16	Specify any planned assessment of meta-bias(es) (such as publication bias across studies, selective reporting within studies)	n/a methodological review
Confidence in cumulative evidence	#17	Describe how the strength of the body of evidence will be assessed (such as GRADE)	n/a methodological review

None The PRISMA-P elaboration and explanation paper is distributed under the terms of the Creative Commons Attribution License CC-BY. This checklist can be completed online using https://www.goodreports.org/, a tool made by the EQUATOR Network in collaboration with Penelope.ai

For peer review only

BMJ Open

Current practices in studies applying the target trial emulation framework: A protocol for a systematic review

Journal:	*BMJ Open*
Manuscript ID	bmjopen-2022-070963.R1
Article Type:	Protocol
Date Submitted by the Author:	13-Apr-2023
Complete List of Authors:	Bigirumurame, Theophile; Newcastle University<br>Hiu, Shaun; Newcastle University<br>Teare, Dawn; Newcastle University<br>Wason, James; Newcastle University<br>Bryant, Andrew; Newcastle University<br>Breckons, Matthew; Newcastle University Institute for Health and Society
**Primary Subject Heading**:	Research methods
Secondary Subject Heading:	Research methods, Evidence based practice
Keywords:	STATISTICS & RESEARCH METHODS, ONCOLOGY, REGISTRIES, Systematic Review

SCHOLARONE™
Manuscripts

TITLE: Current practices in studies applying the target trial emulation framework: A protocol for a systematic review

Short title: Practices in trial emulation

Authors: Theophile Bigirumurame[1], Shaun Kuan Wei Hiu[1], M. Dawn Teare[1], James M. S. Wason[1], Andrew Bryant[1], Matthew Breckons[1]

[1]Population Health Sciences Institute, Newcastle University, UK

Corresponding author:

Theophile Bigirumurame
Population Health Sciences Institutes, Newcastle University,
4th Floor
Ridley Building 1
Queen Victoria Road
Newcastle upon Tyne
NE1 7RU
UK,
Email: theophile.bigirumurame@newcastle.ac.uk
Tel: +44 (0)19120 86776

Authors email addresses: Shaun.Hiu@newcastle.ac.uk, Dawn.Teare@newcastle.ac.uk, James.Wason@newcastle.ac.uk , andy.bryant@newcastle.ac.uk, Matthew.Breckons@newcastle.ac.uk

Abstract:

Introduction: Observational studies represent an alternative to estimate real-world causal effects in the absence of available Randomised Controlled Trials (RCTs). Target trial emulation is a framework for the application of RCT design principles to emulate a hypothetical open-label RCT (the hypothetical target trial) using existing observational data as the primary data source as opposed to the prospective recruitment and measurement of randomised units. The aim of this systematic review is to investigate the practices of studies applying the target trial emulation framework to evaluate the effectiveness of interventions.

Methods and analysis We will systematically search in Medline (via Ovid), Embase (via Ovid, entries from medRxiv are included), PsycINFO (via Ovid), SCOPUS, Web of Science, Cochrane Library, the ISRCTN registry, and ClinicalTrials.gov for all study reports and protocols which used the trial emulation framework (without time restriction). We will extract information concerning study design, data source, analysis, results, interpretation, and dissemination. Two reviewers will perform study selection, data extraction and quality assessment. Disagreements between reviewers will be resolved by a third reviewer. A narrative approach will be used to synthesise and report qualitative and quantitative data. Reporting of the review will be informed by Preferred Reporting Items for Systematic Review and Meta-Analysis guidance (PRISMA).

Ethics and dissemination Ethical approval is not required as it is a protocol for a systematic review. Findings will be disseminated through peer-reviewed publications and conference presentations.

Keywords:
Trial emulation; hypothetical target trial; Target trial emulation; Target trial emulation study, Trial emulation systematic review

Word count= 2315 (excluding abstract, table text, captions, acknowledgment, funding source statement, declaration of interest statement, author contribution statement, and references).

Strengths and limitations

- This systematic review will identify current strengths and weaknesses in how target trial emulation studies are conducted and reported.
- Results may form an evidence-base to inform a bespoke set of reporting guidelines for target trial emulation.
- This systematic review protocol follows the Preferred Reporting Items for Systematic Review and Meta-Analysis Protocols guidelines.
- This review is limited to evidence from published/archived studies
- Exclusion of non-English electronic databases may introduce language bias.

For peer review only - http://bmjopen.bmj.com/site/about/guidelines.xhtml

Introduction

Observational studies represent an alternative to estimate real-world causal effects in the absence of available randomised controlled trials (RCT). Undoubtedly, RCTs are positioned toward the top of the evidence hierarchy in the evaluation of the efficacy and safety of treatments. However, reasons to conduct observational studies instead of RCTs may include better external validity, feasibility, ethical considerations, reduced associated costs, and greater time efficiency [1, 2]. Whilst the use of observational studies to attempt to estimate causal effects is not a new phenomenon – the practice of which dates back as early as Rubin's work [3] on counterfactual theory – the application of RCT design principles to overcome biases in and improve the quality of observational research has been a relatively new development.

Target trial emulation is a framework for the application of RCT design principles to emulate a hypothetical open-label RCT (the hypothetical target trial) using existing observational data as the primary data source as opposed to the prospective recruitment and measurement of randomised units [4]. Because treatment assignment is neither blind nor random, valid causal treatment effects are estimated if the identification principles of causal inference are satisfied [4-8]. These include the assumptions of exchangeability (participants in a study may be swapped between treatment and control groups without changing the average treatment effect), consistency (no multiple versions of the treatment options being investigated), positivity (every patient has a non-zero probability to receive every treatment option under investigation given their measured covariates), and non-interference (the treatment given to one patient does not affect the outcome of another patient) [7, 9-13]. A table of acronyms, abbreviations, and technical terms are defined in appendix.

A study employing target trial emulation is characterised by an explicit description of the hypothetical target trial on several design components: the eligibility criteria, treatment strategy, assignment procedure, follow-up, primary outcome definition, the causal contrast of interest, and a statistical analysis plan [4]. Additionally, an accompanying target trial emulation specification is described and makes explicit how the hypothetical target trial is being emulated along the aforementioned design elements. Stating both the hypothetical target trial and target trial emulation specifications has important benefits. It assists investigators in avoiding biases and pitfalls that are common to the analysis of observational data [14, 15], and permits the sceptical reader to judge if the design of the hypothetical target trial was sensible and if emulation was satisfactory; for example, the eligibility criteria may not be faithfully emulated owing to limitations with the observational data.

Target trial emulation studies can be credible sources of real-world evidence (RWE) to support regulatory decisions in the absence of RCTs, for instance within the Food and Drug Administration's Real-World Evidence Program [16] and the European Medicines Agency's OPerational, TechnIcal, and MethodologicAL framework (OPTIMAL) [17]. Furthermore, with the increasing availability of large-scale real-world data (RWD) especially those seen in

For peer review only - http://bmjopen.bmj.com/site/about/guidelines.xhtml

research cohorts, registry, hospital, and general practitioner databases, there is impetus for increased popularity of the application of target trial emulation.

To our knowledge, no study has reviewed the extant work applying the target trial emulation framework. We argue that a review of this nature is needed for several reasons that have implications for the continued growth of target trial emulation.

First, a systematic review (SR) that reports on the design, conduct, and reporting of target trial emulation studies will help facilitate new research hoping to utilise the methodology. Investigators seeking to conduct their own target trial emulation study may draw guidance from what is currently published, and so a systematic review of the literature serves as a convenient repository of knowledge to new investigators who may be unfamiliar with terminology, choices of designs and analyses, and reporting practices. An additional benefit of the review to both new and experienced investigators is the increased awareness of available datasets that may be pertinent to their field of expertise.

Second, target trial emulation studies attempt to mimic RCTs and thus the Consolidated Standards of Reporting Trials (CONSORT) checklist [18] may be a valid guideline for reporting standards. However, given that target trial emulation studies are necessarily observational studies, the Strengthening the Reporting of Observational Studies in Epidemiology (STROBE) guidelines also apply [19]. Thus, it is essential to capture what (or whether) established reporting guideline(s) have been adopted within the existing trial emulation literature, so that future studies may follow suit. Furthermore, with the recent movement towards the language of the estimand framework in clinical trials [20], it may be useful to capture whether the target trial emulation literature has also displayed such a shift. It may also be that existing checklists are not sufficient to capture all the nuances that exist in target trial emulation. For example, explicit statements on quality assessment such as how the data have been evaluated and judged to be fit-for-purpose for trial emulation are not expected by existing guidelines. Thus, any available reports on data quality may be selective. Clear reporting of these steps is critical especially for regulatory purposes that may require relevance assessments demonstrating fit-for-purpose of the RWD [16]. Additionally, pre-registration is not expected for observational studies but is expected for RCTs. At present there is uncertainty over whether pre-registration should be mandatory, or at least encouraged, for target trial emulation studies.

Third, it may be useful to understand how published studies have discussed the biases and study limitations that preclude perfect emulation of the idealised hypothetical target trial. Recent findings from the Randomized, Controlled Trials Duplicated Using Prospective Longitudinal Insurance Claims: Applying Techniques of Epidemiology (RCT DUPLICATE) initiative compared results of RCTs with their emulation counterparts and found that they do not necessarily agree because perfect emulation may not be possible [21]. This may be due to, but not limited to, differences in treatment adherence rates between RCTs and real-world practice, partial emulation of eligibility criteria due to database limitations, measurement error in the primary outcome, and the challenges associated with emulating a placebo add-on arm. Although not every study applying the target trial emulation framework will be able to make

For peer review only - http://bmjopen.bmj.com/site/about/guidelines.xhtml

comparisons with results from an actual RCT, it is still crucial for authors to postulate on the factors that may strengthen or qualify their interpretation of their results.

Research aims and objectives

The aims and objectives of this SR are to investigate the practices of studies applying the target trial emulation framework to evaluate the risk-benefit of interventions. We aim to examine multiple facets of the study process, specifically pre-specification, study design, data source, analysis, results, interpretation, and dissemination.

Objectives:

1. To review how trial emulation studies have designed their target trial specification (eligibility criteria, treatment strategies, treatment assignment, outcomes, time zero and follow-up, causal contrasts, statistical analysis).
2. To review how the causal inference assumptions were justified.
3. To review pre-registration, reporting, and dissemination practices.
4. To review what study limitations and biases in target trial emulation are commonly discussed.

Methods

Database search

The following electronic databases will be searched: Medline (via Ovid), Embase (via Ovid; entries from medRxiv are included), PsycINFO (via Ovid), SCOPUS, Web of Science, Cochrane Library, the ISRCTN registry, and ClinicalTrials.gov. Search terms for each database are available in Supplementary Table 1. The search will be limited to articles published in English only and no publication date limits will be applied. The start date will be from publication of this protocol. We anticipate a duration of 1.5-years for this systematic review.

Study inclusion and exclusion criteria

Our definition of a target trial emulation study follows from Hernán & Robins [4]:

1. Presence of a hypothetical target trial specification which explicitly describes the trial design components of eligibility criteria, treatment strategy, assignment procedure, follow-up, primary outcome definition, the causal contrast of interest, and a statistical analysis plan; and
2. Presence of a target trial emulation specification which explicitly describes how the hypothetical target trial is being emulated. The specifications may be displayed in tabular format [14] though this need not strictly be the case so long as the information can be retrieved from the text; and
3. Use of at least one observational data source.

Inclusion criteria are as follows: i) meets study definition of target trial emulation study; ii) peer-reviewed reports, protocols (published or archived), study registrations, and conference presentations on original research studies that used/plan to use the trial emulation, from all fields of research; and iii) available in English language. Studies which sought to replicate RCT findings with RWD will also be included since the RCT protocol contains all the information needed in a hypothetical target trial specification and the target trial emulation specification may be inferred from the methods section.

Exclusion criteria are: a) conference abstracts, book chapters, opinions or commentaries, purely qualitative papers, systematic reviews, and meta-analyses; b) conceptual/methodological papers that do not use participant data; and c) non-human subjects.

The reference sections of the excluded systematic reviews, meta-analyses, and methodological papers will be checked to identify eligible papers. Additional articles will also be sourced from studies that cited either Hernán & Robins [4] or a closely-associated paper published in the same year by Hernán, Sauer, Hernández-Díaz, Platt, and Shrier [15].

Selection of studies for inclusion in the review

TB and SKWH will read all titles/and or abstracts retrieved from the database search process and eliminate obviously irrelevant records. The full text of the remaining possibly relevant studies will then be obtained and read, with each record classified as clearly relevant (meets all

For peer review only - http://bmjopen.bmj.com/site/about/guidelines.xhtml

inclusion criteria), clearly irrelevant, or insufficient information to decide. For those cases with insufficient information, corresponding author(s) will be contacted for further information to make a final decision on relevance. In the instance that the full text of a possibly relevant study cannot be retrieved, the corresponding author(s) will be contacted and if no reply is received, the record will be excluded from the final review. Disagreements on eligibility of studies will be arbitrated by a third co-author. References will be managed on Rayyan [22].

Data extraction and management

TB and SKWH will extract data from all included records according to a bespoke data capture form (DCF) developed in Excel and designed around the key research objectives. The DCF will be designed to help review co-authors elicit the required information from the study text with specific questions and prompts. Pre-defined responses will be available for all straightforward yes/no-type questions and certain multiple-choice questions. The multiple-choice questions with pre-defined responses will be those in which the investigators have prior knowledge on a list of possible responses; lists that are judged to be non-exhaustive will be supplemented by an 'Other' response. For questions without this prior knowledge, we will ask the co-author extracting the information to copy the appropriate text verbatim from the paper. The text data will then be subjected to a coding process (see Synthesis of qualitative data section). The extracted data will take the form of close-ended choices (e.g., yes/no and other pre-defined responses), numerical data (e.g., sample size), and qualitative data.

Version 1.0 of the DCF will be developed by adapting information from existing reporting frameworks that are judged to be applicable to target trial emulation. These are the:

1. CONSORT [18];
2. STROBE (combined checklist) [19];
3. REporting of studies Conducted using Observational Routinely-collected health Data (RECORD) [23];
4. The estimand framework [20, 24];
5. Risk Of Bias In Non-randomized Studies – of Interventions (ROBINS-I) assessment tool [25]; and
6. The STRengthening Analytical Thinking for Observational Studies (STRATOS) publications on initial data analysis [26], missing data [27], and causal inference [28].

Version 2.0 will then be piloted on 10 of the included papers and adjustments made where appropriate via discussion with additional authors. Version 3.0 will then be used for all remaining papers. Key study features to be extracted are outlined in Supplementary Table 2; the table functions as a starting point for data collection, which is an evolving process as will be described by version controls of the DCF.

Synthesis of qualitative data

Qualitative data may constitute a mixture of brief commentary or discussion points as well as richer qualitative data describing processes and experiences. These data will be analysed thematically drawing on guidelines for synthesising qualitative research in systematic reviews

For peer review only - http://bmjopen.bmj.com/site/about/guidelines.xhtml

[29] consisting of a three-stage process: 1) TB and SKWH will independently code each line of extracted text based on content and meaning; 2) initial codes will be grouped together according to similarities to form descriptive themes; and 3) analytic themes will be developed based on interpretation of these data in the context of the aims of this review. Each stage of this process will be undertaken by TB and SKWH independently followed by discussion to seek agreement of code and theme generation. Where agreement cannot be reached, an additional co-author will arbitrate.

Summarising and reporting of results

The current protocol adheres to the Preferred Reporting Items for Systematic reviews and Meta-Analyses–Protocol (PRISMA-P) reporting guidelines [30]. Our SR will adhere to the PRISMA reporting guidelines [31, 32].

We will describe the frequency (%) of studies displaying each categorical descriptor of a study feature. For example, when describing types of bias discussed, we may report the frequency and percentage of studies with the following descriptors: "*None stated*", "*Unmeasured confounding*", "*Selection bias*", and so on.

Risk of bias assessment

No risk of bias assessment will be carried out. It is not within the scope of our objectives to render a judgment on whether the hypothetical target trial was successfully emulated or judge if the study results as credible evidence to inform the effectiveness of a treatment. The evidence being synthesised will primarily be the characteristics of the studies, methods, and reporting patterns, as opposed to their results.

Patients and public involvement

No patient involved

Conclusion

This systematic review of target trial emulation studies will give future researchers a detailed overview of how target trial emulation studies have been conducted and reported. Understanding the current strengths and weaknesses of how current studies are being reported will help to improve the design of trial emulation studies and inform bespoke reporting guidelines.

Acknowledgments

We thank Marian Rixham from the Newcastle University Walton library for their expertise in optimising the systematic search strategy.

Contributorship statement

Design of the protocol- TB, SKWH. Draft of the manuscript -TB, SKWH. Review and final approval of the manuscript –TB, SKWH, JMSW, MDT, AB, MB.

Funding statement

TB is funded by the NIHR Applied Research Collaboration North East and North Cumbria (Grant/Award Number: NIHR2001). SKWH (Pre-Doctoral Fellowship, NIHR302746) and JMSW (Research Professorship, NIHR301614) are funded by the National Institute for Health Research (NIHR) for this research project. The views expressed in this publication are those of the author(s) and not necessarily those of the NIHR, National Health Service (NHS), or the UK Department of Health and Social Care.

Conflicts of interest statement

None declared

Data availability statement

Not applicable

Ethical approval

Not required

ORCID:

Theophile Bigirumurame: https://orcid.org/0000-0003-2387-4918

Shaun Kuan Wei Hiu: https://orcid.org/0000-0003-1699-4348

M. Dawn Teare: https://orcid.org/0000-0003-3994-0051

James M. S. Wason: https://orcid.org/0000-0002-4691-126X

Andrew Bryant: https://orcid.org/0000-0003-4351-8865

Matthew Breckons: https://orcid.org/0000-0003-3057-6767

References

1. Kennedy-Martin T, Curtis S, Faries D, Robinson S, Johnston J. A literature review on the representativeness of randomized controlled trial samples and implications for the external validity of trial results. Trials. 2015;16:495.
2. Maringe C, Benitez Majano S, Exarchakou A, Smith M, Rachet B, Belot A, et al. Reflection on modern methods: trial emulation in the presence of immortal-time bias. Assessing the benefit of major surgery for elderly lung cancer patients using observational data. Int J Epidemiol. 2020;49:1719-29.
3. Rubin DE. Estimating causal effects of treatments in randomized and nonrandomized studies. Journal of Educational Psychology. 1974;66:688-701.
4. Hernán MA, Robins JM. Using Big Data to Emulate a Target Trial When a Randomized Trial Is Not Available. American Journal of Epidemiology. 2016;183:758-64.
5. García-Albéniz X, Hsu J, Hernán MA. The value of explicitly emulating a target trial when using real world evidence: an application to colorectal cancer screening. European Journal of Epidemiology. 2017;32:495-500.
6. Hernán MA, Alonso A, Logan R, Grodstein F, Michels KB, Willett WC, et al. Observational studies analyzed like randomized experiments: an application to postmenopausal hormone therapy and coronary heart disease. Epidemiology. 2008;19:766-79.
7. Hernán MA, Robins JM. 3. Observational studies. Causal Inference: What If. Boca Raton: Chapman & Hall/CRC; 2020. p. 27-42.
8. Lodi S, Phillips A, Lundgren J, Logan R, Sharma S, Cole SR, et al. Effect Estimates in Randomized Trials and Observational Studies: Comparing Apples With Apples. Am J Epidemiol. 2019;188:1569-77.
9. Igelström E, Craig P, Lewsey J, Lynch J, Pearce A, Katikireddi SV. Causal inference and effect estimation using observational data. Journal of Epidemiology and Community Health. 2022;76:960.
10. Oakes JM. Effect identification in comparative effectiveness research. EGEMS (Wash DC). 2013;1:1004.
11. Cole SR, Frangakis CE. The consistency statement in causal inference: a definition or an assumption? Epidemiology. 2009;20:3-5.
12. Zhu Y, Hubbard RA, Chubak J, Roy J, Mitra N. Core concepts in pharmacoepidemiology: Violations of the positivity assumption in the causal analysis of observational data: Consequences and statistical approaches. Pharmacoepidemiol Drug Saf. 2021;30:1471-85.
13. Hernán MA. Does water kill? A call for less casual causal inferences. Ann Epidemiol. 2016;26:674-80.
14. Dickerman BA, Garcia-Albeniz X, Logan RW, Denaxas S, Hernán MA. Avoidable flaws in observational analyses: an application to statins and cancer. Nat Med. 2019;25:1601-6.
15. Hernán MA, Sauer BC, Hernandez-Diaz S, Platt R, Shrier I. Specifying a target trial prevents immortal time bias and other self-inflicted injuries in observational analyses. J Clin Epidemiol. 2016;79:70-5.
16. US Food and Drug Administration. Framework for FDA's Real-World Evidence Program. 2018.
17. Cave A, Kurz X, Arlett P. Real-World Data for Regulatory Decision Making: Challenges and Possible Solutions for Europe. Clin Pharmacol Ther. 2019;106:36-9.
18. Schulz KF, Altman DG, Moher D. CONSORT 2010 Statement: Updated Guidelines for Reporting Parallel Group Randomized Trials. Annals of Internal Medicine. 2010;152:726-32.
19. von Elm E, Altman DG, Egger M, Pocock SJ, Gøtzsche PC, Vandenbroucke JP. The Strengthening the Reporting of Observational Studies in Epidemiology (STROBE) Statement: Guidelines for Reporting Observational Studies. Annals of Internal Medicine. 2007;147:573-7.
20. Ratitch B, Bell J, Mallinckrodt C, Bartlett JW, Goel N, Molenberghs G, et al. Choosing Estimands in Clinical Trials: Putting the ICH E9(R1) Into Practice. Ther Innov Regul Sci. 2020;54:324-41.

For peer review only - http://bmjopen.bmj.com/site/about/guidelines.xhtml

21. Franklin JM, Patorno E, Desai RJ, Glynn RJ, Martin D, Quinto K, et al. Emulating Randomized Clinical Trials With Nonrandomized Real-World Evidence Studies: First Results From the RCT DUPLICATE Initiative. Circulation. 2021;143:1002-13.
22. Ouzzani M, Hammady H, Fedorowicz Z, Elmagarmid A. Rayyan-a web and mobile app for systematic reviews. Syst Rev. 2016;5:210.
23. Benchimol EI, Smeeth L, Guttmann A, Harron K, Moher D, Petersen I, et al. The REporting of studies Conducted using Observational Routinely-collected health Data (RECORD) Statement. PLOS Medicine. 2015;12:e1001885.
24. Lipkovich I, Ratitch B, Mallinckrodt CH. Causal Inference and Estimands in Clinical Trials. Statistics in Biopharmaceutical Research. 2020;12:54-67.
25. Sterne JAC, Hernán MA, Reeves BC, Savović J, Berkman ND, Viswanathan M, et al. ROBINS-I: a tool for assessing risk of bias in non-randomised studies of interventions. BMJ. 2016;355:i4919.
26. Huebner M, le Cessie S, Schmidt CO, Vach W. A contemporary conceptual framework for initial data analysis. Observational Studies. 2018;4:171-92.
27. Lee KJ, Tilling KM, Cornish RP, Little RJA, Bell ML, Goetghebeur E, et al. Framework for the treatment and reporting of missing data in observational studies: The Treatment And Reporting of Missing data in Observational Studies framework. J Clin Epidemiol. 2021;134:79-88.
28. Goetghebeur E, le Cessie S, De Stavola B, Moodie EE, Waernbaum I, on behalf of" the topic group Causal Inference of the Si. Formulating causal questions and principled statistical answers. Stat Med. 2020;39:4922-48.
29. Thomas J, Harden A. Methods for the thematic synthesis of qualitative research in systematic reviews. BMC Med Res Methodol. 2008;8:45.
30. Moher D, Shamseer L, Clarke M, Ghersi D, Liberati A, Petticrew M, et al. Preferred reporting items for systematic review and meta-analysis protocols (PRISMA-P) 2015 statement. Syst Rev. 2015;4:1.
31. Moher D, Liberati A, Tetzlaff J, Altman DG, Group P. Preferred reporting items for systematic reviews and meta-analyses: the PRISMA statement. Int J Surg. 2010;8:336-41.
32. Page MJ, McKenzie JE, Bossuyt PM, Boutron I, Hoffmann TC, Mulrow CD, et al. The PRISMA 2020 statement: An updated guideline for reporting systematic reviews. Int J Surg. 2021;88:105906.

For peer review only - http://bmjopen.bmj.com/site/about/guidelines.xhtml

Supplementary Table 1: Search terms

The adjacency search was limited to within six words of the word “trial*”. This was to capture some possible paraphrases of “target trial emulation,” ranging from "trial emulation" to “emulation of a hypothetical randomized controlled trial”. The addition of the (Hernan and Robins) search term is to allow for studies that may have referenced the 2016 paper on the trial emulation framework in the abstract. The ISRCTN registry and ClinicalTrials.gov do not allow for proximity/adjacency searching to the best of our knowledge.

Database	Search terms
Medline (via Ovid)	((duplicat* OR emulat* OR mimic*) ADJ6 trial*).mp OR (Hernan AND Robins).ab
Embase (via Ovid)	Same as above
PsycINFO (via Ovid)	Same as above
SCOPUS	TITLE-ABS-KEY((duplicat* OR emulat* OR mimic*) W/6 trial*) OR ABS(Hernan AND Robins)
Web of Science	((duplicat* OR emulat* OR mimic*) NEAR/6 trial*) (Topic) OR (Hernan AND Robins) (Abstract)
Cochrane Library	(((duplicat* OR emulat* OR mimic*) NEAR/6 trial)):ti,ab,kw OR ((Hernan AND Robins)):ab
ISRCTN registry	"target trial" OR "target trial emulation" OR "trial emulation" OR "emulation of trial" OR "emulation of target trial" OR "emulating a trial" OR "emulating a target trial" OR "emulation of a randomized target trial" OR "emulating a randomized target trial" OR "emulation of a randomized trial" OR "emulating a randomized trial” OR "emulation of a randomized controlled trial" OR "emulating a randomized controlled trial" OR "emulation of RCT" OR "emulating RCT" OR "emulation of a clinical trial" OR "emulating a clinical trial" OR "trial-emulating" OR "hypothetical trial" OR "hypothetical target trial"
ClinicalTrials.gov	"target trial" OR "target trial emulation" OR "trial emulation" OR "emulation of trial" OR "emulation of target trial" OR "emulating a trial" OR "emulating a target trial"

For peer review only

Supplementary Table 2: Key study features to be extracted

Level	Entire study	Target trial emulation framework	
	Target trial emulation study	Hypothetical target trial specification	Target trial emulation specification
Pre-specification	• Pre-registration ID or URL<br>• Pre-registered components: research question; eligibility criteria; treatment strategy; assignment procedure; time zero definition; follow-up period; primary outcome; causal contrasts; statistical analysis plan.		
Research meta	• Research objective<br>• Specialty area<br>• Author<br>• Year of publication<br>• Country		
Data source	• Name of observational data<br>• Type of observational data<br>• Fit-for-purpose assessment		
Target population	• Target population of interest	• Eligibility criteria	• Eligibility criteria
Treatment		• Treatment/therapy<br>• Control/comparator<br>• Grace period from eligibility to treatment initiation	• Treatment/therapy<br>• Control/comparator<br>• Grace period from eligibility to treatment initiation<br>• Time-(in)varying treatment
Assignment procedure		• Hypothetical randomisation strategy	• Method to mimic randomisation<br>• Instrumental variable or confounder selection criteria<br>• Directed acyclic graph
Baseline definition		• Time zero definition	• Time zero definition<br>• Strategy for multiple possible time zeroes<br>• Duration of each "trial's" enrolment period, if strategy is a nested trial design

For peer review only

Follow-up period		• Length<br>• Final visit date<br>• Censoring rules	• Length<br>• Final visit date<br>• Censoring rules
Outcome		• Name of primary outcome<br>• Operational definition	• Name of primary outcome<br>• Operational definition
Causal contrast		• ITT<br>• PP<br>• Estimand framework<br>• Hypothetical inter-current events<br>• Handling inter-current events	• Observational analogue of ITT<br>• Observational analogue of PP<br>• Estimand framework<br>• Observed inter-current events<br>• Handling observed inter-current events
Analysis		• Primary analysis method<br>• Model<br>• Adjustments	• Primary analysis method<br>• Model<br>• Handling of informative censoring<br>• Handling of missing data<br>• Assessment of causal inference assumptions
Results	• Flow diagram		
Discussion	• Design limitations limiting perfect emulation of hypothetical trial<br>• Biases limiting perfect emulation of hypothetical trial<br>• Causal inference assumptions		
Dissemination	• Published in peer-reviewed journal or archived in non-peer reviewed repository<br>• Conference material		
Others	• Reporting guideline(s) used<br>• Deviations from study protocol<br>• Future research recommendations		

For peer review only

Appendix

Key terms, acronyms, and abbreviations

Key term	Description
Hypothetical target trial	The open-label randomised controlled trial that, if conducted, serves as the "gold standard" with regards to evidence for treatment effectiveness.
Target trial emulation	A framework describing the application of randomised controlled trial design principles to emulate the results of a hypothetical target trial using observational data as the primary data source.
Target trial emulation study	A study applying the target trial emulation framework to estimate causal treatment effect(s) and evaluate the risk-benefit of one or more therapies.
Hypothetical target trial specification	An explicit specification of the design of the hypothetical target trial with respect to eligibility criteria, treatment strategy, assignment procedure, follow-up, primary outcome definition, the causal contrast of interest, and a statistical analysis plan.
Target trial emulation specification	An explicit specification of the design of the target trial emulation study that attempts to mimic the hypothetical target trial specification.
Study protocol	A document that details the objectives, design, planned analyses, and reporting procedures of the target trial emulation study as a whole. The study protocol is usually completed before the target trial emulation study is carried out and may be pre-registered.
Potential outcome	An example using a binary observed treatment A and binary observed outcome Y is provided.<br><br>A potential outcome is a *possible* outcome a patient could experience if they were subject to some treatment and is denoted by Y^a, where the lowercase *a* represents the treatment scenario being considered. Thus, $Y^{a=1}$ is the potential outcome of the patient under the scenario had they been given the treatment and $Y^{a=0}$ is the potential outcome of the patient under the scenario had they been given the comparator. A potential outcome then becomes a *realised* outcome once the patient actually receives their treatment. Thus a patient who actually received treatment, their potential becomes realised and their observed outcome is $Y=Y^{a=1}$.
Counterfactual	The version of the potential outcome that is not realised and thus not observed. In essence, we can never observed the patient treated and untreated at the same time.

For peer review only

	Using the example above, because the patient *did* take treatment, the potential outcome $Y^{a=1}$ is now factual and we can observe it, but the potential outcome $Y^{a=0}$ is now counterfactual and we can never observe it.
Exchangeability	One's observed treatment is independent of one's potential outcome. In other words, the risk of the outcome Y=1 in those who received treatment group is the same as those who received the comparator had the patients in the treatment group receive the comparator instead. In practice, this means the relationship between the treatment and the outcome is un-confounded.
Consistency	If one is given treatment option A=a, then one's observed outcome should be the potential outcome under scenario A=a i.e. $Y=Y^a$. In practice, this means the treatment options being investigated are well-defined. Depending on the context, variation within a treatment arm (e.g., different routes of administration) may be acceptable provided these variations are not expected to lead to a different potential outcome.
Positivity	Every patient has a non-zero probability of being given each treatment option under investigation, given their covariates.<br><br>There are two main types of positivity violations Structural positivity may be violated when there is a definite zero probability in some subgroup of patients. For example an investigator includes patients who are contraindicated for one the treatments under investigation. Random positivity violations are situations where whilst it is possible to see variation in treatment within a subgroup, there is no variation in that subgroup in the observed data (e.g., due to sampling variability).
Non-interference	Non-interference requires that a patient's outcome is unaffected (i.e., not interfered) by the treatment given to another patient.
**Acronyms and abbreviations**	**Full title**
CI	Confidence interval
CONSORT	Consolidated Standards of Reporting Trials
EMA	European Medicines Agency
FDA	Food and Drug Administration
HR	Hazard ratio
ICE	Inter-current event
IPTW	Inverse probability of treatment weighting
ITT	Intention-to-treat
OS	Overall survival
PP	Per-protocol

For peer review only

PRISMA	Preferred Reporting Items for Systematic reviews and Meta-Analyses
RCT	Randomised controlled trial
RCT DUPLICATE	Randomized, Controlled Trials Duplicated Using Prospective Longitudinal Insurance Claims: Applying Techniques of Epidemiology
RECORD	REporting of studies Conducted using Observational Routinely-collected health Data
ROBINS-I	Risk Of Bias In Non-randomized Studies – of Interventions
RWD	Real world data
RWE	Real world evidence
SMRW	Standardised morbidity ratio weights
STRATOS	STRengthening Analytical Thinking for Observational Studies
STROBE	Strengthening the Reporting of Observational Studies in Epidemiology
OPTIMAL	OPerational, TechnIcal, and MethodologicAL framework

For peer review only

Reporting checklist for protocol of a systematic review and meta analysis.

Based on the PRISMA-P guidelines.

Instructions to authors

Complete this checklist by entering the page numbers from your manuscript where readers will find each of the items listed below.

Your article may not currently address all the items on the checklist. Please modify your text to include the missing information. If you are certain that an item does not apply, please write "n/a" and provide a short explanation.

Upload your completed checklist as an extra file when you submit to a journal.

In your methods section, say that you used the PRISMA-Preporting guidelines, and cite them as:

Moher D, Shamseer L, Clarke M, Ghersi D, Liberati A, Petticrew M, Shekelle P, Stewart LA. Preferred Reporting Items for Systematic Review and Meta-Analysis Protocols (PRISMA-P) 2015 statement. Syst Rev. 2015;4(1):1.

		Reporting Item	Page Number
**Title**			
Identification	#1a	Identify the report as a protocol of a systematic review	1
Update	#1b	If the protocol is for an update of a previous systematic review, identify as such	n/a

For peer review only

**Registration**			
	#2	If registered, provide the name of the registry (such as PROSPERO) and registration number	n/a methodological review
**Authors**			
Contact	#3a	Provide name, institutional affiliation, e-mail address of all protocol authors; provide physical mailing address of corresponding author	1
Contribution	#3b	Describe contributions of protocol authors and identify the guarantor of the review	8
**Amendments**			
	#4	If the protocol represents an amendment of a previously completed or published protocol, identify as such and list changes; otherwise, state plan for documenting important protocol amendments	n/a first version
**Support**			
Sources	#5a	Indicate sources of financial or other support for the review	9
Sponsor	#5b	Provide name for the review funder and / or sponsor	9
Role of sponsor or funder	#5c	Describe roles of funder(s), sponsor(s), and / or institution(s), if any, in developing the protocol	n/a
**Introduction**			

For peer review only

Rationale	#6	Describe the rationale for the review in the context of what is already known	3-5
Objectives	#7	Provide an explicit statement of the question(s) the review will address with reference to participants, interventions, comparators, and outcomes (PICO)	n/a methodological review
**Methods**			
Eligibility criteria	#8	Specify the study characteristics (such as PICO, study design, setting, time frame) and report characteristics (such as years considered, language, publication status) to be used as criteria for eligibility for the review	6
Information sources	#9	Describe all intended information sources (such as electronic databases, contact with study authors, trial registers or other grey literature sources) with planned dates of coverage	6
Search strategy	#10	Present draft of search strategy to be used for at least one electronic database, including planned limits, such that it could be repeated	6
Study records - data management	#11a	Describe the mechanism(s) that will be used to manage records and data throughout the review	7
Study records - selection process	#11b	State the process that will be used for selecting studies (such as two independent reviewers) through	7

For peer review only

		each phase of the review (that is, screening, eligibility and inclusion in meta-analysis)	
Study records - data collection process	#11c	Describe planned method of extracting data from reports (such as piloting forms, done independently, in duplicate), any processes for obtaining and confirming data from investigators	7
Data items	#12	List and define all variables for which data will be sought (such as PICO items, funding sources), any pre-planned data assumptions and simplifications	6,7
Outcomes and prioritization	#13	List and define all outcomes for which data will be sought, including prioritization of main and additional outcomes, with rationale	n/a methodological review
Risk of bias in individual studies	#14	Describe anticipated methods for assessing risk of bias of individual studies, including whether this will be done at the outcome or study level, or both; state how this information will be used in data synthesis	n/a methodological review
Data synthesis	#15a	Describe criteria under which study data will be quantitatively synthesised	n/a methodological review
Data synthesis	#15b	If data are appropriate for quantitative synthesis, describe planned summary measures, methods of handling data and methods of combining data from studies, including any planned exploration of consistency (such as I2, Kendall's τ)	n/a methodological review

For peer review only

Data synthesis	#15c	Describe any proposed additional analyses (such as sensitivity or subgroup analyses, meta-regression)	n/a methodological review
Data synthesis	#15d	If quantitative synthesis is not appropriate, describe the type of summary planned	Descriptive summary
Meta-bias(es)	#16	Specify any planned assessment of meta-bias(es) (such as publication bias across studies, selective reporting within studies)	n/a methodological review
Confidence in cumulative evidence	#17	Describe how the strength of the body of evidence will be assessed (such as GRADE)	n/a methodological review

None The PRISMA-P elaboration and explanation paper is distributed under the terms of the Creative Commons Attribution License CC-BY. This checklist can be completed online using https://www.goodreports.org/, a tool made by the EQUATOR Network in collaboration with Penelope.ai

For peer review only

BMJ Open

Current practices in studies applying the target trial emulation framework: A protocol for a systematic review

Journal:	*BMJ Open*
Manuscript ID	bmjopen-2022-070963.R2
Article Type:	Protocol
Date Submitted by the Author:	17-May-2023
Complete List of Authors:	Bigirumurame, Theophile; Newcastle University<br>Hiu, Shaun; Newcastle University<br>Teare, Dawn; Newcastle University<br>Wason, James; Newcastle University<br>Bryant, Andrew; Newcastle University<br>Breckons, Matthew; Newcastle University Institute for Health and Society
**Primary Subject Heading**:	Research methods
Secondary Subject Heading:	Research methods, Evidence based practice
Keywords:	STATISTICS & RESEARCH METHODS, ONCOLOGY, REGISTRIES, Systematic Review

SCHOLARONE™
Manuscripts

TITLE: Current practices in studies applying the target trial emulation framework: A protocol for a systematic review

Short title: Practices in trial emulation

Authors: Theophile Bigirumurame[1], Shaun Kuan Wei Hiu[1], M. Dawn Teare[1], James M. S. Wason[1], Andrew Bryant[1], Matthew Breckons[1]

[1]Population Health Sciences Institute, Newcastle University, UK

Corresponding author:

Theophile Bigirumurame
Population Health Sciences Institutes, Newcastle University,
4th Floor
Ridley Building 1
Queen Victoria Road
Newcastle upon Tyne
NE1 7RU
UK,
Email: theophile.bigirumurame@newcastle.ac.uk
Tel: +44 (0)19120 86776

Authors email addresses: Shaun.Hiu@newcastle.ac.uk, Dawn.Teare@newcastle.ac.uk, James.Wason@newcastle.ac.uk , andy.bryant@newcastle.ac.uk, Matthew.Breckons@newcastle.ac.uk

Abstract:

Introduction: Observational studies represent an alternative to estimate real-world causal effects in the absence of available Randomised Controlled Trials (RCTs). Target trial emulation is a framework for the application of RCT design principles to emulate a hypothetical open-label RCT (the hypothetical target trial) using existing observational data as the primary data source as opposed to the prospective recruitment and measurement of randomised units. The aim of this systematic review is to investigate the practices of studies applying the target trial emulation framework to evaluate the effectiveness of interventions.

Methods and analysis We will systematically search in Medline (via Ovid), Embase (via Ovid, entries from medRxiv are included), PsycINFO (via Ovid), SCOPUS, Web of Science, Cochrane Library, the ISRCTN registry, and ClinicalTrials.gov for all study reports and protocols which used the trial emulation framework (without time restriction). We will extract information concerning study design, data source, analysis, results, interpretation, and dissemination. Two reviewers will perform study selection, data extraction and quality assessment. Disagreements between reviewers will be resolved by a third reviewer. A narrative approach will be used to synthesise and report qualitative and quantitative data. Reporting of the review will be informed by Preferred Reporting Items for Systematic Review and Meta-Analysis guidance (PRISMA).

For peer review only - http://bmjopen.bmj.com/site/about/guidelines.xhtml

Ethics and dissemination Ethical approval is not required as it is a protocol for a systematic review. Findings will be disseminated through peer-reviewed publications and conference presentations.

Keywords:
Trial emulation; hypothetical target trial; Target trial emulation; Target trial emulation study, Trial emulation systematic review

Word count= 2315 (excluding abstract, table text, captions, acknowledgment, funding source statement, declaration of interest statement, author contribution statement, and references).

Strengths and limitations

- This systematic review protocol details a comprehensive search strategy consisting of academic databases, Cochrane library, and academic repositories and archives.
- This systematic review protocol pre-specifies qualitative research methods to synthesise extracted text data.
- This systematic review protocol follows the Preferred Reporting Items for Systematic Review and Meta-Analysis Protocols guidelines.
- Risk of bias assessments will not be included in the planned systematic review.
- Exclusion of non-English electronic databases may introduce language bias.

For peer review only - http://bmjopen.bmj.com/site/about/guidelines.xhtml

Introduction

Observational studies represent an alternative to estimate real-world causal effects in the absence of available randomised controlled trials (RCT). Undoubtedly, RCTs are positioned toward the top of the evidence hierarchy in the evaluation of the efficacy and safety of treatments. However, reasons to conduct observational studies instead of RCTs may include better external validity, feasibility, ethical considerations, reduced associated costs, and greater time efficiency [1, 2]. Whilst the use of observational studies to attempt to estimate causal effects is not a new phenomenon – the practice of which dates back as early as Rubin's work [3] on counterfactual theory – the application of RCT design principles to overcome biases in and improve the quality of observational research has been a relatively new development.

Target trial emulation is a framework for the application of RCT design principles to emulate a hypothetical open-label RCT (the hypothetical target trial) using existing observational data as the primary data source as opposed to the prospective recruitment and measurement of randomised units [4]. Because treatment assignment is neither blind nor random, valid causal treatment effects are estimated if the identification principles of causal inference are satisfied [4-8]. These include the assumptions of exchangeability (participants in a study may be swapped between treatment and control groups without changing the average treatment effect), consistency (no multiple versions of the treatment options being investigated), positivity (every patient has a non-zero probability to receive every treatment option under investigation given their measured covariates), and non-interference (the treatment given to one patient does not affect the outcome of another patient) [7, 9-13]. A table of acronyms, abbreviations, and technical terms are defined in appendix.

A study employing target trial emulation is characterised by an explicit description of the hypothetical target trial on several design components: the eligibility criteria, treatment strategy, assignment procedure, follow-up, primary outcome definition, the causal contrast of interest, and a statistical analysis plan [4]. Additionally, an accompanying target trial emulation specification is described and makes explicit how the hypothetical target trial is being emulated along the aforementioned design elements. Stating both the hypothetical target trial and target trial emulation specifications has important benefits. It assists investigators in avoiding biases and pitfalls that are common to the analysis of observational data [14, 15], and permits the sceptical reader to judge if the design of the hypothetical target trial was sensible and if emulation was satisfactory; for example, the eligibility criteria may not be faithfully emulated owing to limitations with the observational data.

Target trial emulation studies can be credible sources of real-world evidence (RWE) to support regulatory decisions in the absence of RCTs, for instance within the Food and Drug Administration's Real-World Evidence Program [16] and the European Medicines Agency's OPerational, TechnIcal, and MethodologicAL framework (OPTIMAL) [17]. Furthermore, with the increasing availability of large-scale real-world data (RWD) especially those seen in

For peer review only - http://bmjopen.bmj.com/site/about/guidelines.xhtml

research cohorts, registry, hospital, and general practitioner databases, there is impetus for increased popularity of the application of target trial emulation.

To our knowledge, no study has reviewed the extant work applying the target trial emulation framework. We argue that a review of this nature is needed for several reasons that have implications for the continued growth of target trial emulation.

First, a systematic review (SR) that reports on the design, conduct, and reporting of target trial emulation studies will help facilitate new research hoping to utilise the methodology. Investigators seeking to conduct their own target trial emulation study may draw guidance from what is currently published, and so a systematic review of the literature serves as a convenient repository of knowledge to new investigators who may be unfamiliar with terminology, choices of designs and analyses, and reporting practices. An additional benefit of the review to both new and experienced investigators is the increased awareness of available datasets that may be pertinent to their field of expertise.

Second, target trial emulation studies attempt to mimic RCTs and thus the Consolidated Standards of Reporting Trials (CONSORT) checklist [18] may be a valid guideline for reporting standards. However, given that target trial emulation studies are necessarily observational studies, the Strengthening the Reporting of Observational Studies in Epidemiology (STROBE) guidelines also apply [19]. Thus, it is essential to capture what (or whether) established reporting guideline(s) have been adopted within the existing trial emulation literature, so that future studies may follow suit. Furthermore, with the recent movement towards the language of the estimand framework in clinical trials [20], it may be useful to capture whether the target trial emulation literature has also displayed such a shift. It may also be that existing checklists are not sufficient to capture all the nuances that exist in target trial emulation. For example, explicit statements on quality assessment such as how the data have been evaluated and judged to be fit-for-purpose for trial emulation are not expected by existing guidelines. Thus, any available reports on data quality may be selective. Clear reporting of these steps is critical especially for regulatory purposes that may require relevance assessments demonstrating fit-for-purpose of the RWD [16]. Additionally, pre-registration is not expected for observational studies but is expected for RCTs. At present there is uncertainty over whether pre-registration should be mandatory, or at least encouraged, for target trial emulation studies.

Third, it may be useful to understand how published studies have discussed the biases and study limitations that preclude perfect emulation of the idealised hypothetical target trial. Recent findings from the Randomized, Controlled Trials Duplicated Using Prospective Longitudinal Insurance Claims: Applying Techniques of Epidemiology (RCT DUPLICATE) initiative compared results of RCTs with their emulation counterparts and found that they do not necessarily agree because perfect emulation may not be possible [21]. This may be due to, but not limited to, differences in treatment adherence rates between RCTs and real-world practice, partial emulation of eligibility criteria due to database limitations, measurement error in the primary outcome, and the challenges associated with emulating a placebo add-on arm. Although not every study applying the target trial emulation framework will be able to make

For peer review only - http://bmjopen.bmj.com/site/about/guidelines.xhtml

comparisons with results from an actual RCT, it is still crucial for authors to postulate on the factors that may strengthen or qualify their interpretation of their results.

Research aims and objectives

The aims and objectives of this SR are to investigate the practices of studies applying the target trial emulation framework to evaluate the risk-benefit of interventions. We aim to examine multiple facets of the study process, specifically pre-specification, study design, data source, analysis, results, interpretation, and dissemination.

Objectives:

1. To review how trial emulation studies have designed their target trial specification (eligibility criteria, treatment strategies, treatment assignment, outcomes, time zero and follow-up, causal contrasts, statistical analysis).
2. To review how the causal inference assumptions were justified.
3. To review pre-registration, reporting, and dissemination practices.
4. To review what study limitations and biases in target trial emulation are commonly discussed.

For peer review only - http://bmjopen.bmj.com/site/about/guidelines.xhtml

Methods

Database search

The following electronic databases will be searched: Medline (via Ovid), Embase (via Ovid; entries from medRxiv are included), PsycINFO (via Ovid), SCOPUS, Web of Science, Cochrane Library, the ISRCTN registry, and ClinicalTrials.gov. Search terms for each database are available in Supplementary Table 1. The search will be limited to articles published in English only and no publication date limits will be applied. We anticipate a duration of 1.5-years for this systematic review (from July 2023 until December 2024).

Study inclusion and exclusion criteria

Our definition of a target trial emulation study follows from Hernán & Robins [4]:

1. Presence of a hypothetical target trial specification which explicitly describes the trial design components of eligibility criteria, treatment strategy, assignment procedure, follow-up, primary outcome definition, the causal contrast of interest, and a statistical analysis plan; and
2. Presence of a target trial emulation specification which explicitly describes how the hypothetical target trial is being emulated. The specifications may be displayed in tabular format [14] though this need not strictly be the case so long as the information can be retrieved from the text; and
3. Used of at least one observational dataset to conduct the trial emulation.

Inclusion criteria are as follows: i) meets study definition of target trial emulation study; ii) peer-reviewed reports, protocols (published or archived), study registrations, and conference presentations on original research studies that used/plan to use the trial emulation, from all fields of research; and iii) available in English language. Studies which sought to replicate RCT findings with RWD will also be included since the RCT protocol contains all the information needed in a hypothetical target trial specification and the target trial emulation specification may be inferred from the methods section.

Exclusion criteria are: a) conference abstracts, book chapters, opinions or commentaries, purely qualitative papers, systematic reviews, and meta-analyses; b) conceptual/methodological papers that do not use participant data; and c) non-human subjects.

The reference sections of the excluded systematic reviews, meta-analyses, and methodological papers will be checked to identify eligible papers. Additional articles will also be sourced from studies that cited either Hernán & Robins [4] or a closely-associated paper published in the same year by Hernán, Sauer, Hernández-Díaz, Platt, and Shrier [15].

Selection of studies for inclusion in the review

TB and SKWH will read all titles/and or abstracts retrieved from the database search process and eliminate obviously irrelevant records. The full text of the remaining possibly relevant studies will then be obtained and read, with each record classified as clearly relevant (meets all

For peer review only - http://bmjopen.bmj.com/site/about/guidelines.xhtml

inclusion criteria), clearly irrelevant, or insufficient information to decide. For those cases with insufficient information, corresponding author(s) will be contacted for further information to make a final decision on relevance. In the instance that the full text of a possibly relevant study cannot be retrieved, the corresponding author(s) will be contacted and if no reply is received, the record will be excluded from the final review. Disagreements on eligibility of studies will be arbitrated by a third co-author. References will be managed on Rayyan [22].

Data extraction and management

TB and SKWH will extract data from all included records according to a bespoke data capture form (DCF) developed in Excel and designed around the key research objectives. The DCF will be designed to help review co-authors elicit the required information from the study text with specific questions and prompts. Pre-defined responses will be available for all straightforward yes/no-type questions and certain multiple-choice questions. The multiple-choice questions with pre-defined responses will be those in which the investigators have prior knowledge on a list of possible responses; lists that are judged to be non-exhaustive will be supplemented by an 'Other' response. For questions without this prior knowledge, we will ask the co-author extracting the information to copy the appropriate text verbatim from the paper. The text data will then be subjected to a coding process (see Synthesis of qualitative data section). The extracted data will take the form of close-ended choices (e.g., yes/no and other pre-defined responses), numerical data (e.g., sample size), and qualitative data.

Version 1.0 of the DCF will be developed by adapting information from existing reporting frameworks that are judged to be applicable to target trial emulation. These are the:

1. CONSORT [18];
2. STROBE (combined checklist) [19];
3. REporting of studies Conducted using Observational Routinely-collected health Data (RECORD) [23];
4. The estimand framework [20, 24];
5. Risk Of Bias In Non-randomized Studies – of Interventions (ROBINS-I) assessment tool [25]; and
6. The STRengthening Analytical Thinking for Observational Studies (STRATOS) publications on initial data analysis [26], missing data [27], and causal inference [28].

Version 1.0 will then be piloted on 10 of the included papers and adjustments made where appropriate via discussion with additional authors (JMSW and MDT). Version 2.0 will then be used for all papers, including the 10 used during the piloting. Key study features to be extracted are outlined in Supplementary Table 2; the table functions as a starting point for data collection, which is an evolving process as will be described by version controls of the DCF.

Synthesis of qualitative data

Qualitative data may constitute a mixture of brief commentary or discussion points as well as richer qualitative data describing processes and experiences. These data will be analysed thematically drawing on guidelines for synthesising qualitative research in systematic reviews

For peer review only - http://bmjopen.bmj.com/site/about/guidelines.xhtml

[29] consisting of a three-stage process: 1) TB and SKWH will independently code each line of extracted text based on content and meaning; 2) initial codes will be grouped together according to similarities to form descriptive themes; and 3) analytic themes will be developed based on interpretation of these data in the context of the aims of this review. Each stage of this process will be undertaken by TB and SKWH independently followed by discussion to seek agreement of code and theme generation. Where agreement cannot be reached, an additional co-author will arbitrate.

Summarising and reporting of results

The current protocol adheres to the Preferred Reporting Items for Systematic reviews and Meta-Analyses–Protocol (PRISMA-P) reporting guidelines [30]. Our SR will adhere to the PRISMA reporting guidelines [31, 32].

We will describe the frequency (%) of studies displaying each categorical descriptor of a study feature. For example, when describing types of bias discussed, we may report the frequency and percentage of studies with the following descriptors: "*None stated*", "*Unmeasured confounding*", "*Selection bias*", and so on.

Risk of bias assessment

No risk of bias assessment will be carried out. It is not within the scope of our objectives to render a judgment on whether the hypothetical target trial was successfully emulated or judge if the study results as credible evidence to inform the effectiveness of a treatment. The evidence being synthesised will primarily be the characteristics of the studies, methods, and reporting patterns, as opposed to their results.

Patients and public involvement

No patient involved

Conclusion

This systematic review of target trial emulation studies will give future researchers a detailed overview of how target trial emulation studies have been conducted and reported. Understanding the current strengths and weaknesses of how current studies are being reported will help to improve the design of trial emulation studies and inform bespoke reporting guidelines.

Acknowledgments

We thank Marian Rixham from the Newcastle University Walton library for their expertise in optimising the systematic search strategy.

Contributorship statement

Design of the protocol- TB, SKWH. Draft of the manuscript -TB, SKWH. Review and final approval of the manuscript –TB, SKWH, JMSW, MDT, AB, MB.

Funding statement

TB is funded by the NIHR Applied Research Collaboration North East and North Cumbria (Grant/Award Number: NIHR2001). SKWH (Pre-Doctoral Fellowship, NIHR302746) and JMSW (Research Professorship, NIHR301614) are funded by the National Institute for Health Research (NIHR) for this research project. The views expressed in this publication are those of the author(s) and not necessarily those of the NIHR, National Health Service (NHS), or the UK Department of Health and Social Care.

Conflicts of interest statement

None declared

Data availability statement

Not applicable

Ethical approval

Not required

ORCID:

Theophile Bigirumurame: https://orcid.org/0000-0003-2387-4918

Shaun Kuan Wei Hiu: https://orcid.org/0000-0003-1699-4348

M. Dawn Teare: https://orcid.org/0000-0003-3994-0051

James M. S. Wason: https://orcid.org/0000-0002-4691-126X

Andrew Bryant: https://orcid.org/0000-0003-4351-8865

Matthew Breckons: https://orcid.org/0000-0003-3057-6767

References

1. Kennedy-Martin T, Curtis S, Faries D, Robinson S, Johnston J. A literature review on the representativeness of randomized controlled trial samples and implications for the external validity of trial results. Trials. 2015;16:495.
2. Maringe C, Benitez Majano S, Exarchakou A, Smith M, Rachet B, Belot A, et al. Reflection on modern methods: trial emulation in the presence of immortal-time bias. Assessing the benefit of major surgery for elderly lung cancer patients using observational data. Int J Epidemiol. 2020;49:1719-29.
3. Rubin DE. Estimating causal effects of treatments in randomized and nonrandomized studies. Journal of Educational Psychology. 1974;66:688-701.
4. Hernán MA, Robins JM. Using Big Data to Emulate a Target Trial When a Randomized Trial Is Not Available. American Journal of Epidemiology. 2016;183:758-64.
5. García-Albéniz X, Hsu J, Hernán MA. The value of explicitly emulating a target trial when using real world evidence: an application to colorectal cancer screening. European Journal of Epidemiology. 2017;32:495-500.
6. Hernán MA, Alonso A, Logan R, Grodstein F, Michels KB, Willett WC, et al. Observational studies analyzed like randomized experiments: an application to postmenopausal hormone therapy and coronary heart disease. Epidemiology. 2008;19:766-79.
7. Hernán MA, Robins JM. 3. Observational studies. Causal Inference: What If. Boca Raton: Chapman & Hall/CRC; 2020. p. 27-42.
8. Lodi S, Phillips A, Lundgren J, Logan R, Sharma S, Cole SR, et al. Effect Estimates in Randomized Trials and Observational Studies: Comparing Apples With Apples. Am J Epidemiol. 2019;188:1569-77.
9. Igelström E, Craig P, Lewsey J, Lynch J, Pearce A, Katikireddi SV. Causal inference and effect estimation using observational data. Journal of Epidemiology and Community Health. 2022;76:960.
10. Oakes JM. Effect identification in comparative effectiveness research. EGEMS (Wash DC). 2013;1:1004.
11. Cole SR, Frangakis CE. The consistency statement in causal inference: a definition or an assumption? Epidemiology. 2009;20:3-5.
12. Zhu Y, Hubbard RA, Chubak J, Roy J, Mitra N. Core concepts in pharmacoepidemiology: Violations of the positivity assumption in the causal analysis of observational data: Consequences and statistical approaches. Pharmacoepidemiol Drug Saf. 2021;30:1471-85.
13. Hernán MA. Does water kill? A call for less casual causal inferences. Ann Epidemiol. 2016;26:674-80.
14. Dickerman BA, Garcia-Albeniz X, Logan RW, Denaxas S, Hernán MA. Avoidable flaws in observational analyses: an application to statins and cancer. Nat Med. 2019;25:1601-6.
15. Hernán MA, Sauer BC, Hernandez-Diaz S, Platt R, Shrier I. Specifying a target trial prevents immortal time bias and other self-inflicted injuries in observational analyses. J Clin Epidemiol. 2016;79:70-5.
16. US Food and Drug Administration. Framework for FDA's Real-World Evidence Program. 2018.
17. Cave A, Kurz X, Arlett P. Real-World Data for Regulatory Decision Making: Challenges and Possible Solutions for Europe. Clin Pharmacol Ther. 2019;106:36-9.
18. Schulz KF, Altman DG, Moher D. CONSORT 2010 Statement: Updated Guidelines for Reporting Parallel Group Randomized Trials. Annals of Internal Medicine. 2010;152:726-32.
19. von Elm E, Altman DG, Egger M, Pocock SJ, Gøtzsche PC, Vandenbroucke JP. The Strengthening the Reporting of Observational Studies in Epidemiology (STROBE) Statement: Guidelines for Reporting Observational Studies. Annals of Internal Medicine. 2007;147:573-7.
20. Ratitch B, Bell J, Mallinckrodt C, Bartlett JW, Goel N, Molenberghs G, et al. Choosing Estimands in Clinical Trials: Putting the ICH E9(R1) Into Practice. Ther Innov Regul Sci. 2020;54:324-41.

For peer review only - http://bmjopen.bmj.com/site/about/guidelines.xhtml

21. Franklin JM, Patorno E, Desai RJ, Glynn RJ, Martin D, Quinto K, et al. Emulating Randomized Clinical Trials With Nonrandomized Real-World Evidence Studies: First Results From the RCT DUPLICATE Initiative. Circulation. 2021;143:1002-13.
22. Ouzzani M, Hammady H, Fedorowicz Z, Elmagarmid A. Rayyan-a web and mobile app for systematic reviews. Syst Rev. 2016;5:210.
23. Benchimol EI, Smeeth L, Guttmann A, Harron K, Moher D, Petersen I, et al. The REporting of studies Conducted using Observational Routinely-collected health Data (RECORD) Statement. PLOS Medicine. 2015;12:e1001885.
24. Lipkovich I, Ratitch B, Mallinckrodt CH. Causal Inference and Estimands in Clinical Trials. Statistics in Biopharmaceutical Research. 2020;12:54-67.
25. Sterne JAC, Hernán MA, Reeves BC, Savović J, Berkman ND, Viswanathan M, et al. ROBINS-I: a tool for assessing risk of bias in non-randomised studies of interventions. BMJ. 2016;355:i4919.
26. Huebner M, le Cessie S, Schmidt CO, Vach W. A contemporary conceptual framework for initial data analysis. Observational Studies. 2018;4:171-92.
27. Lee KJ, Tilling KM, Cornish RP, Little RJA, Bell ML, Goetghebeur E, et al. Framework for the treatment and reporting of missing data in observational studies: The Treatment And Reporting of Missing data in Observational Studies framework. J Clin Epidemiol. 2021;134:79-88.
28. Goetghebeur E, le Cessie S, De Stavola B, Moodie EE, Waernbaum I, on behalf of" the topic group Causal Inference of the Si. Formulating causal questions and principled statistical answers. Stat Med. 2020;39:4922-48.
29. Thomas J, Harden A. Methods for the thematic synthesis of qualitative research in systematic reviews. BMC Med Res Methodol. 2008;8:45.
30. Moher D, Shamseer L, Clarke M, Ghersi D, Liberati A, Petticrew M, et al. Preferred reporting items for systematic review and meta-analysis protocols (PRISMA-P) 2015 statement. Syst Rev. 2015;4:1.
31. Moher D, Liberati A, Tetzlaff J, Altman DG, Group P. Preferred reporting items for systematic reviews and meta-analyses: the PRISMA statement. Int J Surg. 2010;8:336-41.
32. Page MJ, McKenzie JE, Bossuyt PM, Boutron I, Hoffmann TC, Mulrow CD, et al. The PRISMA 2020 statement: An updated guideline for reporting systematic reviews. Int J Surg. 2021;88:105906.

For peer review only - http://bmjopen.bmj.com/site/about/guidelines.xhtml

Supplementary Table 1: Search terms

The adjacency search was limited to within six words of the word “trial*”. This was to capture some possible paraphrases of “target trial emulation,” ranging from "trial emulation" to “emulation of a hypothetical randomized controlled trial”. The addition of the (Hernan and Robins) search term is to allow for studies that may have referenced the 2016 paper on the trial emulation framework in the abstract. The ISRCTN registry and ClinicalTrials.gov do not allow for proximity/adjacency searching to the best of our knowledge.

Database	Search terms
Medline (via Ovid)	((duplicat* OR emulat* OR mimic*) ADJ6 trial*).mp OR (Hernan AND Robins).ab
Embase (via Ovid)	Same as above
PsycINFO (via Ovid)	Same as above
SCOPUS	TITLE-ABS-KEY((duplicat* OR emulat* OR mimic*) W/6 trial*) OR ABS(Hernan AND Robins)
Web of Science	((duplicat* OR emulat* OR mimic*) NEAR/6 trial*) (Topic) OR (Hernan AND Robins) (Abstract)
Cochrane Library	(((duplicat* OR emulat* OR mimic*) NEAR/6 trial)):ti,ab,kw OR ((Hernan AND Robins)):ab
ISRCTN registry	"target trial" OR "target trial emulation" OR "trial emulation" OR "emulation of trial" OR "emulation of target trial" OR "emulating a trial" OR "emulating a target trial" OR "emulation of a randomized target trial" OR "emulating a randomized target trial" OR "emulation of a randomized trial" OR "emulating a randomized trial" OR "emulation of a randomized controlled trial" OR "emulating a randomized controlled trial" OR "emulation of RCT" OR "emulating RCT" OR "emulation of a clinical trial" OR "emulating a clinical trial" OR "trial-emulating" OR "hypothetical trial" OR "hypothetical target trial"
ClinicalTrials.gov	"target trial" OR "target trial emulation" OR "trial emulation" OR "emulation of trial" OR "emulation of target trial" OR "emulating a trial" OR "emulating a target trial"

For peer review only

Supplementary Table 2: Key study features to be extracted

Level	Entire study	Target trial emulation framework	
	Target trial emulation study	Hypothetical target trial specification	Target trial emulation specification
Pre-specification	• Pre-registration ID or URL<br>• Pre-registered components: research question; eligibility criteria; treatment strategy; assignment procedure; time zero definition; follow-up period; primary outcome; causal contrasts; statistical analysis plan.		
Research meta	• Research objective<br>• Specialty area<br>• Author<br>• Year of publication<br>• Country		
Data source	• Name of observational data<br>• Type of observational data<br>• Fit-for-purpose assessment		
Target population	• Target population of interest	• Eligibility criteria	• Eligibility criteria
Treatment		• Treatment/therapy<br>• Control/comparator<br>• Grace period from eligibility to treatment initiation	• Treatment/therapy<br>• Control/comparator<br>• Grace period from eligibility to treatment initiation<br>• Time-(in)varying treatment
Assignment procedure		• Hypothetical randomisation strategy	• Method to mimic randomisation<br>• Instrumental variable or confounder selection criteria<br>• Directed acyclic graph
Baseline definition		• Time zero definition	• Time zero definition<br>• Strategy for multiple possible time zeroes<br>• Duration of each "trial's" enrolment period, if strategy is a nested trial design

For peer review only

Follow-up period		• Length<br>• Final visit date<br>• Censoring rules	• Length<br>• Final visit date<br>• Censoring rules
Outcome		• Name of primary outcome<br>• Operational definition	• Name of primary outcome<br>• Operational definition
Causal contrast		• ITT<br>• PP<br>• Estimand framework<br>• Hypothetical inter-current events<br>• Handling inter-current events	• Observational analogue of ITT<br>• Observational analogue of PP<br>• Estimand framework<br>• Observed inter-current events<br>• Handling observed inter-current events
Analysis		• Primary analysis method<br>• Model<br>• Adjustments	• Primary analysis method<br>• Model<br>• Handling of informative censoring<br>• Handling of missing data<br>• Assessment of causal inference assumptions
Results	• Flow diagram		
Discussion	• Design limitations limiting perfect emulation of hypothetical trial<br>• Biases limiting perfect emulation of hypothetical trial<br>• Causal inference assumptions		
Dissemination	• Published in peer-reviewed journal or archived in non-peer reviewed repository<br>• Conference material		
Others	• Reporting guideline(s) used<br>• Deviations from study protocol<br>• Future research recommendations		

For peer review only

Appendix

Key terms, acronyms, and abbreviations

Key term	Description
Hypothetical target trial	The open-label randomised controlled trial that, if conducted, serves as the "gold standard" with regards to evidence for treatment effectiveness.
Target trial emulation	A framework describing the application of randomised controlled trial design principles to emulate the results of a hypothetical target trial using observational data as the primary data source.
Target trial emulation study	A study applying the target trial emulation framework to estimate causal treatment effect(s) and evaluate the risk-benefit of one or more therapies.
Hypothetical target trial specification	An explicit specification of the design of the hypothetical target trial with respect to eligibility criteria, treatment strategy, assignment procedure, follow-up, primary outcome definition, the causal contrast of interest, and a statistical analysis plan.
Target trial emulation specification	An explicit specification of the design of the target trial emulation study that attempts to mimic the hypothetical target trial specification.
Study protocol	A document that details the objectives, design, planned analyses, and reporting procedures of the target trial emulation study as a whole. The study protocol is usually completed before the target trial emulation study is carried out and may be pre-registered.
Potential outcome	An example using a binary observed treatment A and binary observed outcome Y is provided.<br><br>A potential outcome is a *possible* outcome a patient could experience if they were subject to some treatment and is denoted by Y^a, where the lowercase *a* represents the treatment scenario being considered. Thus, $Y^{a=1}$ is the potential outcome of the patient under the scenario had they been given the treatment and $Y^{a=0}$ is the potential outcome of the patient under the scenario had they been given the comparator. A potential outcome then becomes a *realised* outcome once the patient actually receives their treatment. Thus a patient who actually received treatment, their potential becomes realised and their observed outcome is $Y=Y^{a=1}$.
Counterfactual	The version of the potential outcome that is not realised and thus not observed. In essence, we can never observed the patient treated and untreated at the same time.

	Using the example above, because the patient *did* take treatment, the potential outcome $Y^{a=1}$ is now factual and we can observe it, but the potential outcome $Y^{a=0}$ is now counterfactual and we can never observe it.
Exchangeability	One's observed treatment is independent of one's potential outcome. In other words, the risk of the outcome Y=1 in those who received treatment group is the same as those who received the comparator had the patients in the treatment group receive the comparator instead. In practice, this means the relationship between the treatment and the outcome is un-confounded.
Consistency	If one is given treatment option A=a, then one's observed outcome should be the potential outcome under scenario A=a i.e. $Y=Y^a$. In practice, this means the treatment options being investigated are well-defined. Depending on the context, variation within a treatment arm (e.g., different routes of administration) may be acceptable provided these variations are not expected to lead to a different potential outcome.
Positivity	Every patient has a non-zero probability of being given each treatment option under investigation, given their covariates.<br><br>There are two main types of positivity violations Structural positivity may be violated when there is a definite zero probability in some subgroup of patients. For example an investigator includes patients who are contraindicated for one the treatments under investigation. Random positivity violations are situations where whilst it is possible to see variation in treatment within a subgroup, there is no variation in that subgroup in the observed data (e.g., due to sampling variability).
Non-interference	Non-interference requires that a patient's outcome is unaffected (i.e., not interfered) by the treatment given to another patient.
**Acronyms and abbreviations**	**Full title**
CI	Confidence interval
CONSORT	Consolidated Standards of Reporting Trials
EMA	European Medicines Agency
FDA	Food and Drug Administration
HR	Hazard ratio
ICE	Inter-current event
IPTW	Inverse probability of treatment weighting
ITT	Intention-to-treat
OS	Overall survival
PP	Per-protocol

For peer review only - http://bmjopen.bmj.com/site/about/guidelines.xhtml

PRISMA	Preferred Reporting Items for Systematic reviews and Meta-Analyses
RCT	Randomised controlled trial
RCT DUPLICATE	Randomized, Controlled Trials Duplicated Using Prospective Longitudinal Insurance Claims: Applying Techniques of Epidemiology
RECORD	REporting of studies Conducted using Observational Routinely-collected health Data
ROBINS-I	Risk Of Bias In Non-randomized Studies – of Interventions
RWD	Real world data
RWE	Real world evidence
SMRW	Standardised morbidity ratio weights
STRATOS	STRengthening Analytical Thinking for Observational Studies
STROBE	Strengthening the Reporting of Observational Studies in Epidemiology
OPTIMAL	OPerational, TechnIcal, and MethodologicAL framework

For peer review only

Reporting checklist for protocol of a systematic review and meta analysis.

Based on the PRISMA-P guidelines.

Instructions to authors

Complete this checklist by entering the page numbers from your manuscript where readers will find each of the items listed below.

Your article may not currently address all the items on the checklist. Please modify your text to include the missing information. If you are certain that an item does not apply, please write "n/a" and provide a short explanation.

Upload your completed checklist as an extra file when you submit to a journal.

In your methods section, say that you used the PRISMA-Preporting guidelines, and cite them as:

Moher D, Shamseer L, Clarke M, Ghersi D, Liberati A, Petticrew M, Shekelle P, Stewart LA. Preferred Reporting Items for Systematic Review and Meta-Analysis Protocols (PRISMA-P) 2015 statement. Syst Rev. 2015;4(1):1.

		Reporting Item	Page Number
**Title**			
Identification	#1a	Identify the report as a protocol of a systematic review	1
Update	#1b	If the protocol is for an update of a previous systematic review, identify as such	n/a

For peer review only

**Registration**			
	#2	If registered, provide the name of the registry (such as PROSPERO) and registration number	n/a methodological review
**Authors**			
Contact	#3a	Provide name, institutional affiliation, e-mail address of all protocol authors; provide physical mailing address of corresponding author	1
Contribution	#3b	Describe contributions of protocol authors and identify the guarantor of the review	8
**Amendments**			
	#4	If the protocol represents an amendment of a previously completed or published protocol, identify as such and list changes; otherwise, state plan for documenting important protocol amendments	n/a first version
**Support**			
Sources	#5a	Indicate sources of financial or other support for the review	9
Sponsor	#5b	Provide name for the review funder and / or sponsor	9
Role of sponsor or funder	#5c	Describe roles of funder(s), sponsor(s), and / or institution(s), if any, in developing the protocol	n/a
**Introduction**			

For peer review only

Rationale	#6	Describe the rationale for the review in the context of what is already known	3-5
Objectives	#7	Provide an explicit statement of the question(s) the review will address with reference to participants, interventions, comparators, and outcomes (PICO)	n/a methodological review
**Methods**			
Eligibility criteria	#8	Specify the study characteristics (such as PICO, study design, setting, time frame) and report characteristics (such as years considered, language, publication status) to be used as criteria for eligibility for the review	6
Information sources	#9	Describe all intended information sources (such as electronic databases, contact with study authors, trial registers or other grey literature sources) with planned dates of coverage	6
Search strategy	#10	Present draft of search strategy to be used for at least one electronic database, including planned limits, such that it could be repeated	6
Study records - data management	#11a	Describe the mechanism(s) that will be used to manage records and data throughout the review	7
Study records - selection process	#11b	State the process that will be used for selecting studies (such as two independent reviewers) through	7

For peer review only

		each phase of the review (that is, screening, eligibility and inclusion in meta-analysis)	
Study records - data collection process	#11c	Describe planned method of extracting data from reports (such as piloting forms, done independently, in duplicate), any processes for obtaining and confirming data from investigators	7
Data items	#12	List and define all variables for which data will be sought (such as PICO items, funding sources), any pre-planned data assumptions and simplifications	6,7
Outcomes and prioritization	#13	List and define all outcomes for which data will be sought, including prioritization of main and additional outcomes, with rationale	n/a methodological review
Risk of bias in individual studies	#14	Describe anticipated methods for assessing risk of bias of individual studies, including whether this will be done at the outcome or study level, or both; state how this information will be used in data synthesis	n/a methodological review
Data synthesis	#15a	Describe criteria under which study data will be quantitatively synthesised	n/a methodological review
Data synthesis	#15b	If data are appropriate for quantitative synthesis, describe planned summary measures, methods of handling data and methods of combining data from studies, including any planned exploration of consistency (such as I2, Kendall's τ)	n/a methodological review

For peer review only

Data synthesis	#15c	Describe any proposed additional analyses (such as sensitivity or subgroup analyses, meta-regression)	n/a methodological review
Data synthesis	#15d	If quantitative synthesis is not appropriate, describe the type of summary planned	Descriptive summary
Meta-bias(es)	#16	Specify any planned assessment of meta-bias(es) (such as publication bias across studies, selective reporting within studies)	n/a methodological review
Confidence in cumulative evidence	#17	Describe how the strength of the body of evidence will be assessed (such as GRADE)	n/a methodological review

None The PRISMA-P elaboration and explanation paper is distributed under the terms of the Creative Commons Attribution License CC-BY. This checklist can be completed online using https://www.goodreports.org/, a tool made by the EQUATOR Network in collaboration with Penelope.ai

For peer review only

BMJ Open

Current practices in studies applying the target trial emulation framework: A protocol for a systematic review

Journal:	*BMJ Open*
Manuscript ID	bmjopen-2022-070963.R3
Article Type:	Protocol
Date Submitted by the Author:	02-Jun-2023

Complete List of Authors:	Bigirumurame, Theophile; Newcastle University<br>Hiu, Shaun; Newcastle University<br>Teare, Dawn; Newcastle University<br>Wason, James; Newcastle University<br>Bryant, Andrew; Newcastle University<br>Breckons, Matthew; Newcastle University Institute for Health and Society

**Primary Subject Heading**:	Research methods
Secondary Subject Heading:	Research methods, Evidence based practice
Keywords:	STATISTICS & RESEARCH METHODS, ONCOLOGY, REGISTRIES, Systematic Review

SCHOLARONE™ Manuscripts

TITLE: Current practices in studies applying the target trial emulation framework: A protocol for a systematic review

Short title: Practices in trial emulation

Authors: Theophile Bigirumurame[1], Shaun Kuan Wei Hiu[1], M. Dawn Teare[1], James M. S. Wason[1], Andrew Bryant[1], Matthew Breckons[1]

[1]Population Health Sciences Institute, Newcastle University, UK

Corresponding author:

Theophile Bigirumurame
Population Health Sciences Institutes, Newcastle University,
4th Floor
Ridley Building 1
Queen Victoria Road
Newcastle upon Tyne
NE1 7RU
UK,
Email: theophile.bigirumurame@newcastle.ac.uk
Tel: +44 (0)19120 86776

Authors email addresses: Shaun.Hiu@newcastle.ac.uk, Dawn.Teare@newcastle.ac.uk, James.Wason@newcastle.ac.uk , andy.bryant@newcastle.ac.uk, Matthew.Breckons@newcastle.ac.uk

Abstract:

Introduction: Observational studies represent an alternative to estimate real-world causal effects in the absence of available Randomised Controlled Trials (RCTs). Target trial emulation is a framework for the application of RCT design principles to emulate a hypothetical open-label RCT (the hypothetical target trial) using existing observational data as the primary data source as opposed to the prospective recruitment and measurement of randomised units. The aim of this systematic review is to investigate the practices of studies applying the target trial emulation framework to evaluate the effectiveness of interventions.

Methods and analysis We will systematically search in Medline (via Ovid), Embase (via Ovid, entries from medRxiv are included), PsycINFO (via Ovid), SCOPUS, Web of Science, Cochrane Library, the ISRCTN registry, and ClinicalTrials.gov for all study reports and protocols which used the trial emulation framework (without time restriction). We will extract information concerning study design, data source, analysis, results, interpretation, and dissemination. Two reviewers will perform study selection, data extraction and quality assessment. Disagreements between reviewers will be resolved by a third reviewer. A narrative approach will be used to synthesise and report qualitative and quantitative data. Reporting of the review will be informed by Preferred Reporting Items for Systematic Review and Meta-Analysis guidance (PRISMA).

Ethics and dissemination Ethical approval is not required as it is a protocol for a systematic review. Findings will be disseminated through peer-reviewed publications and conference presentations.

Keywords:
Trial emulation; hypothetical target trial; Target trial emulation; Target trial emulation study, Trial emulation systematic review

Word count= 2315 (excluding abstract, table text, captions, acknowledgment, funding source statement, declaration of interest statement, author contribution statement, and references).

Strengths and limitations

- This systematic review protocol details a comprehensive search strategy consisting of academic databases, Cochrane library, and academic repositories and archives.
- This systematic review protocol pre-specifies qualitative research methods to synthesise extracted text data.
- This systematic review protocol follows the Preferred Reporting Items for Systematic Review and Meta-Analysis Protocols guidelines.
- Risk of bias assessments will not be included in the planned systematic review.
- Exclusion of non-English electronic databases may introduce language bias.

Introduction

Observational studies represent an alternative to estimate real-world causal effects in the absence of available randomised controlled trials (RCT). Undoubtedly, RCTs are positioned toward the top of the evidence hierarchy in the evaluation of the efficacy and safety of treatments. However, reasons to conduct observational studies instead of RCTs may include better external validity, feasibility, ethical considerations, reduced associated costs, and greater time efficiency [1, 2]. Whilst the use of observational studies to attempt to estimate causal effects is not a new phenomenon – the practice of which dates back as early as Rubin's work [3] on counterfactual theory – the application of RCT design principles to overcome biases in and improve the quality of observational research has been a relatively new development.

Target trial emulation is a framework for the application of RCT design principles to emulate a hypothetical open-label RCT (the hypothetical target trial) using existing observational data as the primary data source as opposed to the prospective recruitment and measurement of randomised units [4]. Because treatment assignment is neither blind nor random, valid causal treatment effects are estimated if the identification principles of causal inference are satisfied [4-8]. These include the assumptions of exchangeability (participants in a study may be swapped between treatment and control groups without changing the average treatment effect), consistency (no multiple versions of the treatment options being investigated), positivity (every patient has a non-zero probability to receive every treatment option under investigation given their measured covariates), and non-interference (the treatment given to one patient does not affect the outcome of another patient) [7, 9-13]. A table of acronyms, abbreviations, and technical terms are defined in appendix.

A study employing target trial emulation is characterised by an explicit description of the hypothetical target trial on several design components: the eligibility criteria, treatment strategy, assignment procedure, follow-up, primary outcome definition, the causal contrast of interest, and a statistical analysis plan [4]. Additionally, an accompanying target trial emulation specification is described and makes explicit how the hypothetical target trial is being emulated along the aforementioned design elements. Stating both the hypothetical target trial and target trial emulation specifications has important benefits. It assists investigators in avoiding biases and pitfalls that are common to the analysis of observational data [14, 15], and permits the sceptical reader to judge if the design of the hypothetical target trial was sensible and if emulation was satisfactory; for example, the eligibility criteria may not be faithfully emulated owing to limitations with the observational data.

Target trial emulation studies can be credible sources of real-world evidence (RWE) to support regulatory decisions in the absence of RCTs, for instance within the Food and Drug Administration's Real-World Evidence Program [16] and the European Medicines Agency's OPerational, TechnIcal, and MethodologicAL framework (OPTIMAL) [17]. Furthermore, with the increasing availability of large-scale real-world data (RWD) especially those seen in

For peer review only - http://bmjopen.bmj.com/site/about/guidelines.xhtml

research cohorts, registry, hospital, and general practitioner databases, there is impetus for increased popularity of the application of target trial emulation.

To our knowledge, no study has reviewed the extant work applying the target trial emulation framework. We argue that a review of this nature is needed for several reasons that have implications for the continued growth of target trial emulation.

First, a systematic review (SR) that reports on the design, conduct, and reporting of target trial emulation studies will help facilitate new research hoping to utilise the methodology. Investigators seeking to conduct their own target trial emulation study may draw guidance from what is currently published, and so a systematic review of the literature serves as a convenient repository of knowledge to new investigators who may be unfamiliar with terminology, choices of designs and analyses, and reporting practices. An additional benefit of the review to both new and experienced investigators is the increased awareness of available datasets that may be pertinent to their field of expertise.

Second, target trial emulation studies attempt to mimic RCTs and thus the Consolidated Standards of Reporting Trials (CONSORT) checklist [18] may be a valid guideline for reporting standards. However, given that target trial emulation studies are necessarily observational studies, the Strengthening the Reporting of Observational Studies in Epidemiology (STROBE) guidelines also apply [19]. Thus, it is essential to capture what (or whether) established reporting guideline(s) have been adopted within the existing trial emulation literature, so that future studies may follow suit. Furthermore, with the recent movement towards the language of the estimand framework in clinical trials [20], it may be useful to capture whether the target trial emulation literature has also displayed such a shift. It may also be that existing checklists are not sufficient to capture all the nuances that exist in target trial emulation. For example, explicit statements on quality assessment such as how the data have been evaluated and judged to be fit-for-purpose for trial emulation are not expected by existing guidelines. Thus, any available reports on data quality may be selective. Clear reporting of these steps is critical especially for regulatory purposes that may require relevance assessments demonstrating fit-for-purpose of the RWD [16]. Additionally, pre-registration is not expected for observational studies but is expected for RCTs. At present there is uncertainty over whether pre-registration should be mandatory, or at least encouraged, for target trial emulation studies.

Third, it may be useful to understand how published studies have discussed the biases and study limitations that preclude perfect emulation of the idealised hypothetical target trial. Recent findings from the Randomized, Controlled Trials Duplicated Using Prospective Longitudinal Insurance Claims: Applying Techniques of Epidemiology (RCT DUPLICATE) initiative compared results of RCTs with their emulation counterparts and found that they do not necessarily agree because perfect emulation may not be possible [21]. This may be due to, but not limited to, differences in treatment adherence rates between RCTs and real-world practice, partial emulation of eligibility criteria due to database limitations, measurement error in the primary outcome, and the challenges associated with emulating a placebo add-on arm. Although not every study applying the target trial emulation framework will be able to make

For peer review only - http://bmjopen.bmj.com/site/about/guidelines.xhtml

comparisons with results from an actual RCT, it is still crucial for authors to postulate on the factors that may strengthen or qualify their interpretation of their results.

Research aims and objectives

The aims and objectives of this SR are to investigate the practices of studies applying the target trial emulation framework to evaluate the risk-benefit of interventions. We aim to examine multiple facets of the study process, specifically pre-specification, study design, data source, analysis, results, interpretation, and dissemination.

Objectives:

1. To review how trial emulation studies have designed their target trial specification (eligibility criteria, treatment strategies, treatment assignment, outcomes, time zero and follow-up, causal contrasts, statistical analysis).
2. To review how the causal inference assumptions were justified.
3. To review pre-registration, reporting, and dissemination practices.
4. To review what study limitations and biases in target trial emulation are commonly discussed.

Methods

Database search

The following electronic databases will be searched: Medline (via Ovid), Embase (via Ovid; entries from medRxiv are included), PsycINFO (via Ovid), SCOPUS, Web of Science, Cochrane Library, the ISRCTN registry, and ClinicalTrials.gov. Search terms for each database are available in Supplementary Table 1. The search will be limited to articles published in English only and no publication date limits will be applied. We anticipate a duration of 1.5-years for this systematic review (from July 2023 until December 2024).

Study inclusion and exclusion criteria

Eligible studies must first meet the definition of a target trial emulation study based on Hernán & Robins [4]:

1. Presence of a hypothetical target trial specification which explicitly describes the trial design components of eligibility criteria, treatment strategy, assignment procedure, follow-up, primary outcome definition, the causal contrast of interest, and a statistical analysis plan; and
2. Presence of a target trial emulation specification which explicitly describes how the hypothetical target trial is being emulated. The specifications may be displayed in tabular format [14] though this need not strictly be the case so long as the information can be retrieved from the text; and
3. The study must have used of at least one observational dataset to conduct their trial emulation.

Additional inclusion criteria are as follows: i) meets study definition of target trial emulation study; ii) peer-reviewed reports, protocols (published or archived), study registrations, and conference presentations on original research studies that used/plan to use the trial emulation, from all fields of research; and iii) available in English language. Studies which sought to replicate RCT findings with RWD will also be included since the RCT protocol contains all the information needed in a hypothetical target trial specification and the target trial emulation specification may be inferred from the methods section.

Exclusion criteria are: a) conference abstracts, book chapters, opinions or commentaries, purely qualitative papers, systematic reviews, and meta-analyses; b) conceptual/methodological papers that do not use participant data; and c) non-human subjects.

The reference sections of the excluded systematic reviews, meta-analyses, and methodological papers will be checked to identify eligible papers. Additional articles will also be sourced from studies that cited either Hernán & Robins [4] or a closely-associated paper published in the same year by Hernán, Sauer, Hernández-Díaz, Platt, and Shrier [15].

Selection of studies for inclusion in the review

TB and SKWH will read all titles/and or abstracts retrieved from the database search process and eliminate obviously irrelevant records. The full text of the remaining possibly relevant studies will then be obtained and read, with each record classified as clearly relevant (meets all inclusion criteria), clearly irrelevant, or insufficient information to decide. For those cases with insufficient information, corresponding author(s) will be contacted for further information to make a final decision on relevance. In the instance that the full text of a possibly relevant study cannot be retrieved, the corresponding author(s) will be contacted and if no reply is received, the record will be excluded from the final review. Disagreements on eligibility of studies will be arbitrated by a third co-author. References will be managed on Rayyan [22].

Data extraction and management

TB and SKWH will extract data from all included records according to a bespoke data capture form (DCF) developed in Excel and designed around the key research objectives. The DCF will be designed to help review co-authors elicit the required information from the study text with specific questions and prompts. Pre-defined responses will be available for all straightforward yes/no-type questions and certain multiple-choice questions. The multiple-choice questions with pre-defined responses will be those in which the investigators have prior knowledge on a list of possible responses; lists that are judged to be non-exhaustive will be supplemented by an 'Other' response. For questions without this prior knowledge, we will ask the co-author extracting the information to copy the appropriate text verbatim from the paper. The text data will then be subjected to a coding process (see Synthesis of qualitative data section). The extracted data will take the form of close-ended choices (e.g., yes/no and other pre-defined responses), numerical data (e.g., sample size), and qualitative data.

Version 1.0 of the DCF will be developed by adapting information from existing reporting frameworks that are judged to be applicable to target trial emulation. These are the:

1. CONSORT [18];
2. STROBE (combined checklist) [19];
3. REporting of studies Conducted using Observational Routinely-collected health Data (RECORD) [23];
4. The estimand framework [20, 24];
5. Risk Of Bias In Non-randomized Studies – of Interventions (ROBINS-I) assessment tool [25]; and
6. The STRengthening Analytical Thinking for Observational Studies (STRATOS) publications on initial data analysis [26], missing data [27], and causal inference [28].

Version 1.0 will then be piloted on 10 of the included papers and adjustments made where appropriate via discussion with additional authors (JMSW and MDT). Version 2.0 will then be used for all papers, including the 10 used during the piloting. Key study features to be extracted are outlined in Supplementary Table 2; the table functions as a starting point for data collection, which is an evolving process as will be described by version controls of the DCF.

Synthesis of qualitative data

For peer review only - http://bmjopen.bmj.com/site/about/guidelines.xhtml

Qualitative data may constitute a mixture of brief commentary or discussion points as well as richer qualitative data describing processes and experiences. These data will be analysed thematically drawing on guidelines for synthesising qualitative research in systematic reviews [29] consisting of a three-stage process: 1) TB and SKWH will independently code each line of extracted text based on content and meaning; 2) initial codes will be grouped together according to similarities to form descriptive themes; and 3) analytic themes will be developed based on interpretation of these data in the context of the aims of this review. Each stage of this process will be undertaken by TB and SKWH independently followed by discussion to seek agreement of code and theme generation. Where agreement cannot be reached, an additional co-author will arbitrate.

Summarising and reporting of results

The current protocol adheres to the Preferred Reporting Items for Systematic reviews and Meta-Analyses–Protocol (PRISMA-P) reporting guidelines [30]. Our SR will adhere to the PRISMA reporting guidelines [31, 32].

We will describe the frequency (%) of studies displaying each categorical descriptor of a study feature. For example, when describing types of bias discussed, we may report the frequency and percentage of studies with the following descriptors: "*None stated*", "*Unmeasured confounding*", "*Selection bias*", and so on.

Risk of bias assessment

No risk of bias assessment will be carried out. It is not within the scope of our objectives to render a judgment on whether the hypothetical target trial was successfully emulated or judge if the study results as credible evidence to inform the effectiveness of a treatment. The evidence being synthesised will primarily be the characteristics of the studies, methods, and reporting patterns, as opposed to their results.

Patients and public involvement

No patient involved

Conclusion

This systematic review of target trial emulation studies will give future researchers a detailed overview of how target trial emulation studies have been conducted and reported. Understanding the current strengths and weaknesses of how current studies are being reported will help to improve the design of trial emulation studies and inform bespoke reporting guidelines.

Acknowledgments

We thank Marian Rixham from the Newcastle University Walton library for their expertise in optimising the systematic search strategy.

Contributorship statement

Design of the protocol- TB, SKWH. Draft of the manuscript -TB, SKWH. Review and final approval of the manuscript –TB, SKWH, JMSW, MDT, AB, MB.

Funding statement

TB is funded by the NIHR Applied Research Collaboration North East and North Cumbria (Grant/Award Number: NIHR2001). SKWH (Pre-Doctoral Fellowship, NIHR302746) and JMSW (Research Professorship, NIHR301614) are funded by the National Institute for Health Research (NIHR) for this research project. The views expressed in this publication are those of the author(s) and not necessarily those of the NIHR, National Health Service (NHS), or the UK Department of Health and Social Care.

Conflicts of interest statement

None declared

Data availability statement

Not applicable

Ethical approval

Not required

ORCID:

Theophile Bigirumurame: https://orcid.org/0000-0003-2387-4918

Shaun Kuan Wei Hiu: https://orcid.org/0000-0003-1699-4348

M. Dawn Teare: https://orcid.org/0000-0003-3994-0051

James M. S. Wason: https://orcid.org/0000-0002-4691-126X

Andrew Bryant: https://orcid.org/0000-0003-4351-8865

Matthew Breckons: https://orcid.org/0000-0003-3057-6767

References

1. Kennedy-Martin T, Curtis S, Faries D, Robinson S, Johnston J. A literature review on the representativeness of randomized controlled trial samples and implications for the external validity of trial results. Trials. 2015;16:495.
2. Maringe C, Benitez Majano S, Exarchakou A, Smith M, Rachet B, Belot A, et al. Reflection on modern methods: trial emulation in the presence of immortal-time bias. Assessing the benefit of major surgery for elderly lung cancer patients using observational data. Int J Epidemiol. 2020;49:1719-29.
3. Rubin DE. Estimating causal effects of treatments in randomized and nonrandomized studies. Journal of Educational Psychology. 1974;66:688-701.
4. Hernán MA, Robins JM. Using Big Data to Emulate a Target Trial When a Randomized Trial Is Not Available. American Journal of Epidemiology. 2016;183:758-64.
5. García-Albéniz X, Hsu J, Hernán MA. The value of explicitly emulating a target trial when using real world evidence: an application to colorectal cancer screening. European Journal of Epidemiology. 2017;32:495-500.
6. Hernán MA, Alonso A, Logan R, Grodstein F, Michels KB, Willett WC, et al. Observational studies analyzed like randomized experiments: an application to postmenopausal hormone therapy and coronary heart disease. Epidemiology. 2008;19:766-79.
7. Hernán MA, Robins JM. 3. Observational studies. Causal Inference: What If. Boca Raton: Chapman & Hall/CRC; 2020. p. 27-42.
8. Lodi S, Phillips A, Lundgren J, Logan R, Sharma S, Cole SR, et al. Effect Estimates in Randomized Trials and Observational Studies: Comparing Apples With Apples. Am J Epidemiol. 2019;188:1569-77.
9. Igelström E, Craig P, Lewsey J, Lynch J, Pearce A, Katikireddi SV. Causal inference and effect estimation using observational data. Journal of Epidemiology and Community Health. 2022;76:960.
10. Oakes JM. Effect identification in comparative effectiveness research. EGEMS (Wash DC). 2013;1:1004.
11. Cole SR, Frangakis CE. The consistency statement in causal inference: a definition or an assumption? Epidemiology. 2009;20:3-5.
12. Zhu Y, Hubbard RA, Chubak J, Roy J, Mitra N. Core concepts in pharmacoepidemiology: Violations of the positivity assumption in the causal analysis of observational data: Consequences and statistical approaches. Pharmacoepidemiol Drug Saf. 2021;30:1471-85.
13. Hernán MA. Does water kill? A call for less casual causal inferences. Ann Epidemiol. 2016;26:674-80.
14. Dickerman BA, Garcia-Albeniz X, Logan RW, Denaxas S, Hernán MA. Avoidable flaws in observational analyses: an application to statins and cancer. Nat Med. 2019;25:1601-6.
15. Hernán MA, Sauer BC, Hernandez-Diaz S, Platt R, Shrier I. Specifying a target trial prevents immortal time bias and other self-inflicted injuries in observational analyses. J Clin Epidemiol. 2016;79:70-5.
16. US Food and Drug Administration. Framework for FDA's Real-World Evidence Program. 2018.
17. Cave A, Kurz X, Arlett P. Real-World Data for Regulatory Decision Making: Challenges and Possible Solutions for Europe. Clin Pharmacol Ther. 2019;106:36-9.
18. Schulz KF, Altman DG, Moher D. CONSORT 2010 Statement: Updated Guidelines for Reporting Parallel Group Randomized Trials. Annals of Internal Medicine. 2010;152:726-32.
19. von Elm E, Altman DG, Egger M, Pocock SJ, Gøtzsche PC, Vandenbroucke JP. The Strengthening the Reporting of Observational Studies in Epidemiology (STROBE) Statement: Guidelines for Reporting Observational Studies. Annals of Internal Medicine. 2007;147:573-7.
20. Ratitch B, Bell J, Mallinckrodt C, Bartlett JW, Goel N, Molenberghs G, et al. Choosing Estimands in Clinical Trials: Putting the ICH E9(R1) Into Practice. Ther Innov Regul Sci. 2020;54:324-41.

For peer review only - http://bmjopen.bmj.com/site/about/guidelines.xhtml

21. Franklin JM, Patorno E, Desai RJ, Glynn RJ, Martin D, Quinto K, et al. Emulating Randomized Clinical Trials With Nonrandomized Real-World Evidence Studies: First Results From the RCT DUPLICATE Initiative. Circulation. 2021;143:1002-13.
22. Ouzzani M, Hammady H, Fedorowicz Z, Elmagarmid A. Rayyan-a web and mobile app for systematic reviews. Syst Rev. 2016;5:210.
23. Benchimol EI, Smeeth L, Guttmann A, Harron K, Moher D, Petersen I, et al. The REporting of studies Conducted using Observational Routinely-collected health Data (RECORD) Statement. PLOS Medicine. 2015;12:e1001885.
24. Lipkovich I, Ratitch B, Mallinckrodt CH. Causal Inference and Estimands in Clinical Trials. Statistics in Biopharmaceutical Research. 2020;12:54-67.
25. Sterne JAC, Hernán MA, Reeves BC, Savović J, Berkman ND, Viswanathan M, et al. ROBINS-I: a tool for assessing risk of bias in non-randomised studies of interventions. BMJ. 2016;355:i4919.
26. Huebner M, le Cessie S, Schmidt CO, Vach W. A contemporary conceptual framework for initial data analysis. Observational Studies. 2018;4:171-92.
27. Lee KJ, Tilling KM, Cornish RP, Little RJA, Bell ML, Goetghebeur E, et al. Framework for the treatment and reporting of missing data in observational studies: The Treatment And Reporting of Missing data in Observational Studies framework. J Clin Epidemiol. 2021;134:79-88.
28. Goetghebeur E, le Cessie S, De Stavola B, Moodie EE, Waernbaum I, on behalf of" the topic group Causal Inference of the Si. Formulating causal questions and principled statistical answers. Stat Med. 2020;39:4922-48.
29. Thomas J, Harden A. Methods for the thematic synthesis of qualitative research in systematic reviews. BMC Med Res Methodol. 2008;8:45.
30. Moher D, Shamseer L, Clarke M, Ghersi D, Liberati A, Petticrew M, et al. Preferred reporting items for systematic review and meta-analysis protocols (PRISMA-P) 2015 statement. Syst Rev. 2015;4:1.
31. Moher D, Liberati A, Tetzlaff J, Altman DG, Group P. Preferred reporting items for systematic reviews and meta-analyses: the PRISMA statement. Int J Surg. 2010;8:336-41.
32. Page MJ, McKenzie JE, Bossuyt PM, Boutron I, Hoffmann TC, Mulrow CD, et al. The PRISMA 2020 statement: An updated guideline for reporting systematic reviews. Int J Surg. 2021;88:105906.

For peer review only - http://bmjopen.bmj.com/site/about/guidelines.xhtml

Supplementary Table 1: Search terms

The adjacency search was limited to within six words of the word “trial*”. This was to capture some possible paraphrases of “target trial emulation,” ranging from "trial emulation" to “emulation of a hypothetical randomized controlled trial”. The addition of the (Hernan and Robins) search term is to allow for studies that may have referenced the 2016 paper on the trial emulation framework in the abstract. The ISRCTN registry and ClinicalTrials.gov do not allow for proximity/adjacency searching to the best of our knowledge.

**Database**	**Search terms**
Medline (via Ovid)	((duplicat* OR emulat* OR mimic*) ADJ6 trial*).mp OR (Hernan AND Robins).ab
Embase (via Ovid)	Same as above
PsycINFO (via Ovid)	Same as above
SCOPUS	TITLE-ABS-KEY((duplicat* OR emulat* OR mimic*) W/6 trial*) OR ABS(Hernan AND Robins)
Web of Science	((duplicat* OR emulat* OR mimic*) NEAR/6 trial*) (Topic) OR (Hernan AND Robins) (Abstract)
Cochrane Library	(((duplicat* OR emulat* OR mimic*) NEAR/6 trial)):ti,ab,kw OR ((Hernan AND Robins)):ab
ISRCTN registry	"target trial" OR "target trial emulation" OR "trial emulation" OR "emulation of trial" OR "emulation of target trial" OR "emulating a trial" OR "emulating a target trial" OR "emulation of a randomized target trial" OR "emulating a randomized target trial" OR "emulation of a randomized trial" OR "emulating a randomized trial” OR "emulation of a randomized controlled trial" OR "emulating a randomized controlled trial" OR "emulation of RCT" OR "emulating RCT" OR "emulation of a clinical trial" OR "emulating a clinical trial" OR "trial-emulating" OR "hypothetical trial" OR "hypothetical target trial"
ClinicalTrials.gov	"target trial" OR "target trial emulation" OR "trial emulation" OR "emulation of trial" OR "emulation of target trial" OR "emulating a trial" OR "emulating a target trial"

For peer review only

Supplementary Table 2: Key study features to be extracted

Level	Entire study	Target trial emulation framework	
	Target trial emulation study	Hypothetical target trial specification	Target trial emulation specification
Pre-specification	• Pre-registration ID or URL<br>• Pre-registered components: research question; eligibility criteria; treatment strategy; assignment procedure; time zero definition; follow-up period; primary outcome; causal contrasts; statistical analysis plan.		
Research meta	• Research objective<br>• Specialty area<br>• Author<br>• Year of publication<br>• Country		
Data source	• Name of observational data<br>• Type of observational data<br>• Fit-for-purpose assessment		
Target population	• Target population of interest	• Eligibility criteria	• Eligibility criteria
Treatment		• Treatment/therapy<br>• Control/comparator<br>• Grace period from eligibility to treatment initiation	• Treatment/therapy<br>• Control/comparator<br>• Grace period from eligibility to treatment initiation<br>• Time-(in)varying treatment
Assignment procedure		• Hypothetical randomisation strategy	• Method to mimic randomisation<br>• Instrumental variable or confounder selection criteria<br>• Directed acyclic graph
Baseline definition		• Time zero definition	• Time zero definition<br>• Strategy for multiple possible time zeroes<br>• Duration of each “trial’s” enrolment period, if strategy is a nested trial design

For peer review only

Follow-up period		• Length<br>• Final visit date<br>• Censoring rules	• Length<br>• Final visit date<br>• Censoring rules
Outcome		• Name of primary outcome<br>• Operational definition	• Name of primary outcome<br>• Operational definition
Causal contrast		• ITT<br>• PP<br>• Estimand framework<br>• Hypothetical inter-current events<br>• Handling inter-current events	• Observational analogue of ITT<br>• Observational analogue of PP<br>• Estimand framework<br>• Observed inter-current events<br>• Handling observed inter-current events
Analysis		• Primary analysis method<br>• Model<br>• Adjustments	• Primary analysis method<br>• Model<br>• Handling of informative censoring<br>• Handling of missing data<br>• Assessment of causal inference assumptions
Results	• Flow diagram		
Discussion	• Design limitations limiting perfect emulation of hypothetical trial<br>• Biases limiting perfect emulation of hypothetical trial<br>• Causal inference assumptions		
Dissemination	• Published in peer-reviewed journal or archived in non-peer reviewed repository<br>• Conference material		
Others	• Reporting guideline(s) used<br>• Deviations from study protocol<br>• Future research recommendations		

For peer review only

Appendix

Key terms, acronyms, and abbreviations

Key term	Description
Hypothetical target trial	The open-label randomised controlled trial that, if conducted, serves as the "gold standard" with regards to evidence for treatment effectiveness.
Target trial emulation	A framework describing the application of randomised controlled trial design principles to emulate the results of a hypothetical target trial using observational data as the primary data source.
Target trial emulation study	A study applying the target trial emulation framework to estimate causal treatment effect(s) and evaluate the risk-benefit of one or more therapies.
Hypothetical target trial specification	An explicit specification of the design of the hypothetical target trial with respect to eligibility criteria, treatment strategy, assignment procedure, follow-up, primary outcome definition, the causal contrast of interest, and a statistical analysis plan.
Target trial emulation specification	An explicit specification of the design of the target trial emulation study that attempts to mimic the hypothetical target trial specification.
Study protocol	A document that details the objectives, design, planned analyses, and reporting procedures of the target trial emulation study as a whole. The study protocol is usually completed before the target trial emulation study is carried out and may be pre-registered.
Potential outcome	An example using a binary observed treatment A and binary observed outcome Y is provided.<br><br>A potential outcome is a *possible* outcome a patient could experience if they were subject to some treatment and is denoted by Y^a, where the lowercase *a* represents the treatment scenario being considered. Thus, $Y^{a=1}$ is the potential outcome of the patient under the scenario had they been given the treatment and $Y^{a=0}$ is the potential outcome of the patient under the scenario had they been given the comparator. A potential outcome then becomes a *realised* outcome once the patient actually receives their treatment. Thus a patient who actually received treatment, their potential becomes realised and their observed outcome is $Y=Y^{a=1}$.
Counterfactual	The version of the potential outcome that is not realised and thus not observed. In essence, we can never observed the patient treated and untreated at the same time.

For peer review only

	Using the example above, because the patient *did* take treatment, the potential outcome $Y^{a=1}$ is now factual and we can observe it, but the potential outcome $Y^{a=0}$ is now counterfactual and we can never observe it.
Exchangeability	One's observed treatment is independent of one's potential outcome. In other words, the risk of the outcome Y=1 in those who received treatment group is the same as those who received the comparator had the patients in the treatment group receive the comparator instead. In practice, this means the relationship between the treatment and the outcome is un-confounded.
Consistency	If one is given treatment option A=a, then one's observed outcome should be the potential outcome under scenario A=a i.e. $Y=Y^a$. In practice, this means the treatment options being investigated are well-defined. Depending on the context, variation within a treatment arm (e.g., different routes of administration) may be acceptable provided these variations are not expected to lead to a different potential outcome.
Positivity	Every patient has a non-zero probability of being given each treatment option under investigation, given their covariates.<br><br>There are two main types of positivity violations Structural positivity may be violated when there is a definite zero probability in some subgroup of patients. For example an investigator includes patients who are contraindicated for one the treatments under investigation. Random positivity violations are situations where whilst it is possible to see variation in treatment within a subgroup, there is no variation in that subgroup in the observed data (e.g., due to sampling variability).
Non-interference	Non-interference requires that a patient's outcome is unaffected (i.e., not interfered) by the treatment given to another patient.
**Acronyms and abbreviations**	**Full title**
CI	Confidence interval
CONSORT	Consolidated Standards of Reporting Trials
EMA	European Medicines Agency
FDA	Food and Drug Administration
HR	Hazard ratio
ICE	Inter-current event
IPTW	Inverse probability of treatment weighting
ITT	Intention-to-treat
OS	Overall survival
PP	Per-protocol

For peer review only

PRISMA	Preferred Reporting Items for Systematic reviews and Meta-Analyses
RCT	Randomised controlled trial
RCT DUPLICATE	Randomized, Controlled Trials Duplicated Using Prospective Longitudinal Insurance Claims: Applying Techniques of Epidemiology
RECORD	REporting of studies Conducted using Observational Routinely-collected health Data
ROBINS-I	Risk Of Bias In Non-randomized Studies – of Interventions
RWD	Real world data
RWE	Real world evidence
SMRW	Standardised morbidity ratio weights
STRATOS	STRengthening Analytical Thinking for Observational Studies
STROBE	Strengthening the Reporting of Observational Studies in Epidemiology
OPTIMAL	OPerational, TechnIcal, and MethodologicAL framework

For peer review only

Reporting checklist for protocol of a systematic review and meta analysis.

Based on the PRISMA-P guidelines.

Instructions to authors

Complete this checklist by entering the page numbers from your manuscript where readers will find each of the items listed below.

Your article may not currently address all the items on the checklist. Please modify your text to include the missing information. If you are certain that an item does not apply, please write "n/a" and provide a short explanation.

Upload your completed checklist as an extra file when you submit to a journal.

In your methods section, say that you used the PRISMA-Preporting guidelines, and cite them as:

Moher D, Shamseer L, Clarke M, Ghersi D, Liberati A, Petticrew M, Shekelle P, Stewart LA. Preferred Reporting Items for Systematic Review and Meta-Analysis Protocols (PRISMA-P) 2015 statement. Syst Rev. 2015;4(1):1.

		Reporting Item	Page Number
**Title**			
Identification	#1a	Identify the report as a protocol of a systematic review	1
Update	#1b	If the protocol is for an update of a previous systematic review, identify as such	n/a

For peer review only - http://bmjopen.bmj.com/site/about/guidelines.xhtml

**Registration**			
	#2	If registered, provide the name of the registry (such as PROSPERO) and registration number	n/a methodological review
**Authors**			
Contact	#3a	Provide name, institutional affiliation, e-mail address of all protocol authors; provide physical mailing address of corresponding author	1
Contribution	#3b	Describe contributions of protocol authors and identify the guarantor of the review	8
**Amendments**			
	#4	If the protocol represents an amendment of a previously completed or published protocol, identify as such and list changes; otherwise, state plan for documenting important protocol amendments	n/a first version
**Support**			
Sources	#5a	Indicate sources of financial or other support for the review	9
Sponsor	#5b	Provide name for the review funder and / or sponsor	9
Role of sponsor or funder	#5c	Describe roles of funder(s), sponsor(s), and / or institution(s), if any, in developing the protocol	n/a
**Introduction**			

For peer review only

Rationale	#6	Describe the rationale for the review in the context of what is already known	3-5
Objectives	#7	Provide an explicit statement of the question(s) the review will address with reference to participants, interventions, comparators, and outcomes (PICO)	n/a methodological review
**Methods**			
Eligibility criteria	#8	Specify the study characteristics (such as PICO, study design, setting, time frame) and report characteristics (such as years considered, language, publication status) to be used as criteria for eligibility for the review	6
Information sources	#9	Describe all intended information sources (such as electronic databases, contact with study authors, trial registers or other grey literature sources) with planned dates of coverage	6
Search strategy	#10	Present draft of search strategy to be used for at least one electronic database, including planned limits, such that it could be repeated	6
Study records - data management	#11a	Describe the mechanism(s) that will be used to manage records and data throughout the review	7
Study records - selection process	#11b	State the process that will be used for selecting studies (such as two independent reviewers) through	7

For peer review only

		each phase of the review (that is, screening, eligibility and inclusion in meta-analysis)	
Study records - data collection process	#11c	Describe planned method of extracting data from reports (such as piloting forms, done independently, in duplicate), any processes for obtaining and confirming data from investigators	7
Data items	#12	List and define all variables for which data will be sought (such as PICO items, funding sources), any pre-planned data assumptions and simplifications	6,7
Outcomes and prioritization	#13	List and define all outcomes for which data will be sought, including prioritization of main and additional outcomes, with rationale	n/a methodological review
Risk of bias in individual studies	#14	Describe anticipated methods for assessing risk of bias of individual studies, including whether this will be done at the outcome or study level, or both; state how this information will be used in data synthesis	n/a methodological review
Data synthesis	#15a	Describe criteria under which study data will be quantitatively synthesised	n/a methodological review
Data synthesis	#15b	If data are appropriate for quantitative synthesis, describe planned summary measures, methods of handling data and methods of combining data from studies, including any planned exploration of consistency (such as I2, Kendall's τ)	n/a methodological review

For peer review only

Data synthesis	#15c	Describe any proposed additional analyses (such as sensitivity or subgroup analyses, meta-regression)	n/a methodological review
Data synthesis	#15d	If quantitative synthesis is not appropriate, describe the type of summary planned	Descriptive summary
Meta-bias(es)	#16	Specify any planned assessment of meta-bias(es) (such as publication bias across studies, selective reporting within studies)	n/a methodological review
Confidence in cumulative evidence	#17	Describe how the strength of the body of evidence will be assessed (such as GRADE)	n/a methodological review

None The PRISMA-P elaboration and explanation paper is distributed under the terms of the Creative Commons Attribution License CC-BY. This checklist can be completed online using https://www.goodreports.org/, a tool made by the EQUATOR Network in collaboration with Penelope.ai

For peer review only
